# Comparative analysis of pre- and post-parasitic transcriptomes and mining pioneer effectors of *Heterodera avenae*

**DOI:** 10.1186/s13578-017-0138-6

**Published:** 2017-02-14

**Authors:** Dan Yang, Changlong Chen, Qian Liu, Heng Jian

**Affiliations:** 1 Department of Plant Pathology, China Agricultural University, Beijing, 100193 China; 2 Institute of Crop Research, Chinese Academy of Agricultural Sciences, Beijing, 100081 China

**Keywords:** *Heterodera avenae*, Pre-parasitic and post-parasitic stage, Transcriptome sequencing, Effectors, Comparative analysis

## Abstract

**Background:**

The cereal cyst nematode (CCN, *Heterodera avenae*) is a devastating pathogen of wheat and barley crops in many countries. We aimed to prioritize genetic and molecular targets for *H. avenae* control via the powerful and integrative bioinformatics platform.

**Results:**

Here, we sequenced mRNA isolated from Chinese *H. avenae* at pre-parasitic (consisting of egg, J1 and hatched-J2) stages and post-parasitic (consisting of parasitic-J2, J3, J4 and adults) stages. Total 1,066,719 reads of whole life cycle transcriptomes were assembled into 10,811 contigs with N50 length of 1754 bp and 71,401 singletons. Comparative analyses of orthologous among *H. avenae* and 7 other nematodes with various life-styles revealed the significance and peculiarity of neurological system for sedentary phytonematode. KEGG pathway enrichment demonstrated active crosstalk events of nervous system at pre-parasitic stages, and 6 FMRFamide-like neuropeptides were verified to display an expression peak at the hatched-J2 stage in *H. avenae*. Furthermore, multiple approaches were undertaken to mine putative effectors and parasitism-specific genes. Notably, *H. avenae* might represent the first phytonematode reported to possess the pioneer effectors with RxLR motif and potential effectors with homologies to *Ant*-*5/Ant*-*34*.

**Conclusion:**

Our work provides valuable resources for in-depth understanding the parasitism and pathogenicity of *H. avenae,* as well as developing new targets-oriented strategies on effective managements.

**Electronic supplementary material:**

The online version of this article (doi:10.1186/s13578-017-0138-6) contains supplementary material, which is available to authorized users.

## Background

As a worldwide plant pathogen, the cereal cyst nematode (CCN) *Heterodera avenae* is an essential threat to global food security. It is widely distributed in more or less temperate wheat-producing regions throughout the world; in some wheat fields, the losses caused by this nematode range from 30 to 100% [1, 2]. In China, the occurrence of *H. avenae* has distributed in 16 provinces, covering 80% of the total wheat growing regions (approximately 20 million ha), and has been reducing the yield in significant proportions every year [3].

*Heterodera avenae* is an obligate, sedentary endoparasite that has unique, biotrophic interactions with its host plants and reproduces by amphimixis. Its life cycle can be divided into pre-parasitic stages and post-parasitic stages. Life cycle of *H. avenae* starts with an egg present inside an encysted female. In pre-parasitic stages, the first stage larva (J1) develops within the egg, and the second stage juvenile (J2) hatches from cyst and migrates into the soil. Upon localizing host plant roots, the parasitic J2 invades the host tissue by its stylet and migrates intracellularly to select initial feeding cell for subsequent establishment of syncytial feeding site [4, 5]. Maintaining the enlarged syncytium is essential for the permanent nutrient sink during the sedentary J3 and J4 stages. It takes 3–6 weeks for developing into an adult male or an egg-laying female.

Based on the life style, the managements of *H. avenae* target at either pre-parasitic stages or post-parasitic stages. However, particular problems are caused by the ability of encysted *H. avenae* to survive for prolonged periods in the soil in the absence of a host, making control by rotations difficult [5]. Additionally, resistant cultivar is limited by the lack of dominant natural resistance genes and the long breeding period. Moreover, the use of chemical nematicides is withdrawn or severely limited by recent legislation due to potential adverse effects to both human health and natural environment [6]. Therefore, exploring novel approaches based on new gene targets is urgently needed for control of *H. avenae*.

Effectors, as ‘all pathogen proteins and small molecules that alter host-cell structure and function’, are considered valuable targets for novel management strategies of parasitic nematodes [7]. It is generally accepted that secretions produced by *H. avenae* are responsible for the successful parasitism. Particularly, the effectors expressed exclusively in the esophageal gland cells and secreted through the stylet play key roles in this process [8, 9]. Thus, effective strategies to discover effectors are presumed to rely on identifying nematode secretions directly by cDNA sequencing or proteomics approaches [10–15]. However, a more common and high throughput approach is to investigate the transcriptome or genome via bioinformatics tools to predict large putatively secreted proteins that are not limited to those from esophageal gland cells [16–19].

Recently, Kumar et al. reported the first transcriptomic analysis of two life stages of Indian population of *H. avenae*, including hatched-J2 and feeding female, and provided a friendly resource of database (HATdb) to help seeking the effectors [20]. However, the critical parasitism processes such as penetration, migration and feeding site initiation, establishment and maintenance were carried out from the early reaction of parasitism and during the life-long time pathogenesis of cyst nematodes [21]. For instance, the expression peaks of suppressors (*Gr*-UBCEP12 [22] and *Gr*-SPRYSEC-4/5/15/18/19 proteins [23]) to the plant defense response were present at parasitic J2 or J3 stage in potato cyst nematode *Globodera rostochiensis*. The perception of nematode CLAVATA3 (CLV3)/ESR (CLE)-like proteins by CLV2 and CRN was detected at the nematode-induced syncytia when the beet cyst nematode *Heterodera schachtii* developed into the parasitic-J2, J3 or J4 stages [24]. Thus, the genetic data of partial life stages might miss some effectors with low abundance or unique functions for suppressing the primary plant defense response or inducing and maintaining the syncytia by *H. avenae* during parasitism. More recently, with a focus on the early parasitic J2 stage (30 h, 3 and 9 days post infection), Zheng et al. reported 56 putative effectors among 681 parasitism genes of *H. avenae* during incompatible infection to *Aegilops variabilis* [25]. Nevertheless, it was arduous to fully separate the parasitic nematode genes from the plant-nematode mixed transcriptomes. Moreover, current available *H. avenae* gene set is not complete enough to analyze the whole life cycle of nematode invasion. Besides, these analyses might also lack genes specifically expressed in cyst stage to ensure the long-term surviving in the soil. Therefore, further expansion of the genetic resources of *H. avenae* and identification of pivotal parasitism genes as well as specific regulatory pathways of its whole life cycle were required.

In this study, we have sequenced transcriptomes of the pre-parasitic stages (including eggs, J1 and hatched-J2) and post-parasitic stages (including parasitic-J2, J3, J4, and adults) of Chinese *H. avenae*. In the context of other published nematode genetic datasets, we undertook multiple comparative analyses among nematodes with different life styles. Specific ESTs expressed in *H. avenae* were identified, and gene transcripts profiles have been compared between pre- and post-parasitic stages. Putative effectors, including pioneer effectors with RxLR motif and other potential effectors in *H. avenae*, were found through multiple strategies. These effectors highlighted important in-depth insights into the pivotal genes involved in suppressing plant defense and regulation of host plants for successful parasitism. Our work benefits the comprehensive elucidation of the molecular mechanisms underling parasitism and pathogenicity of *H. avenae*, and also provides valuable information on potential gene targets for its control.

## Results

### General overview of *H. avenae* transcriptome sequencing, assembly and annotations

**Fig. 1:**
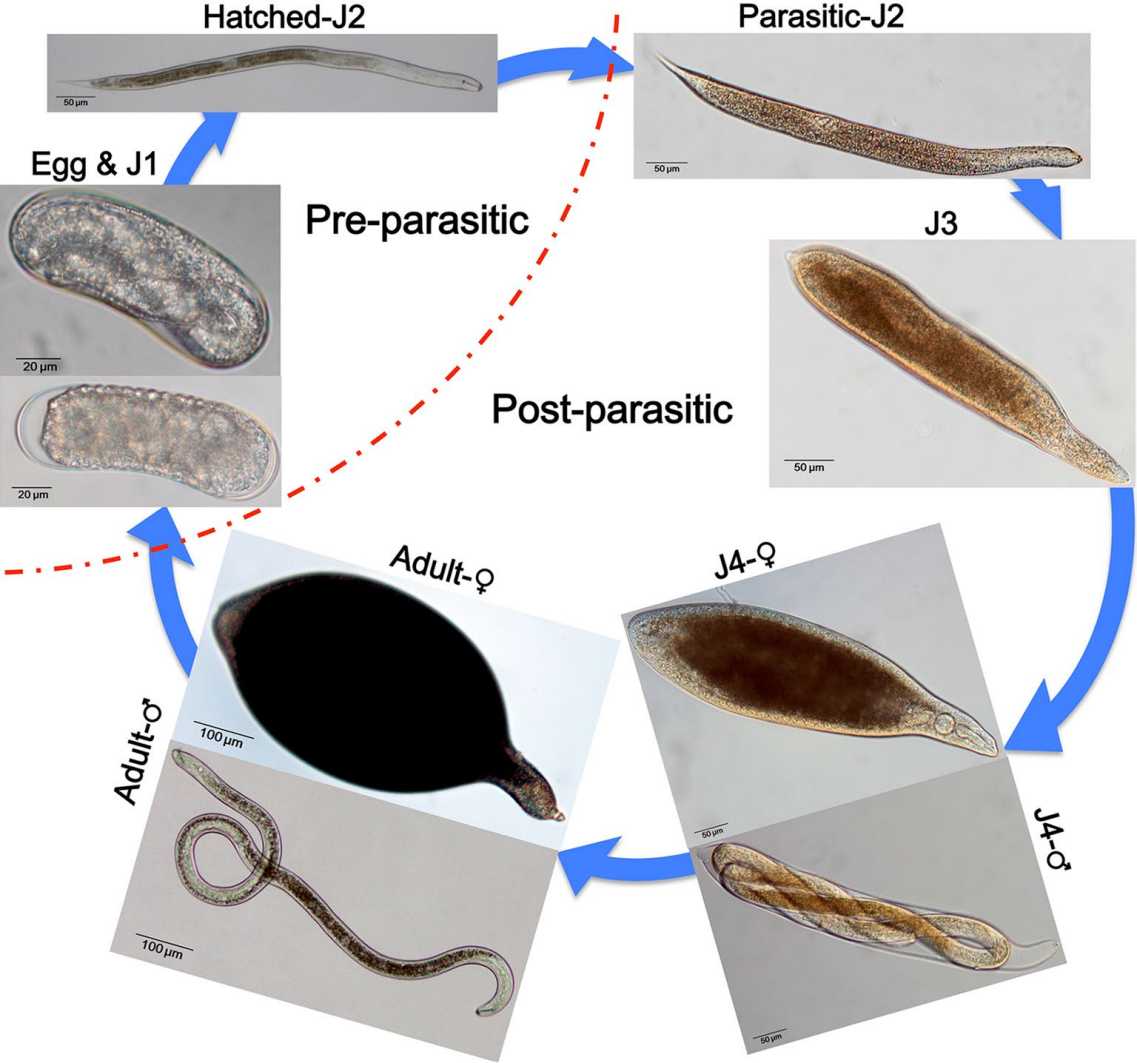
Developmental stages of *Heterodera avenae* sampled for transcriptomic and gene expression analysis. Chinese *H. avenae* at pre-parasitic stages consisted of egg, J1 and hatched-J2 nematodes (on the *up*-*left* of the *dotted curve*) and post-parasitic stages consisted of parasitic-J2, J3, J4 and adult nematodes (on the *down*-*right* of the *dotted curve*)

**Fig. 2:**
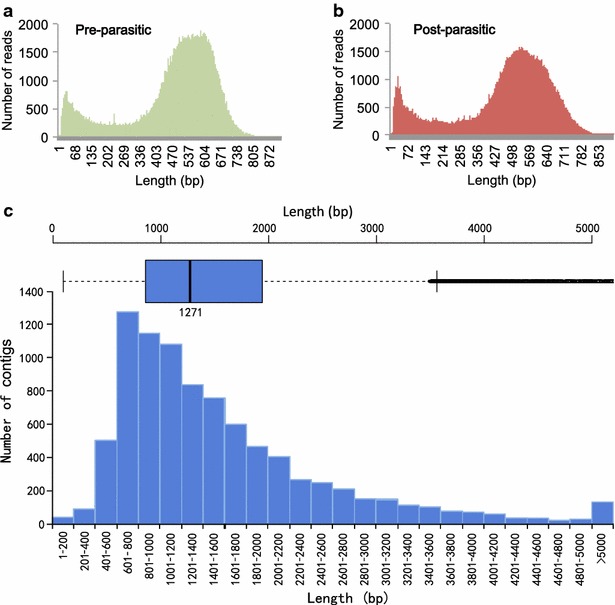
The distribution of sequences lengths in the transcriptomes of *H. avenae* by 454 FLX+ sequencing. *Histogram graphs* show the distribution of reads lengths for the transcriptomes of *H. avenae* at pre-parasitic stages (**a**) and post-parasitic stages (**b**). **c** *Bar graph* shows the distribution of contigs lengths from the assembled transcriptome of whole life cycle *H. avenae* covering the above-mentioned two mixed stages

Transcriptome sequencing using 454 FLX+/Roche platform was performed for pre-parasitic and post-parasitic stages of Chinese *H. avenae* (Fig. 1). This yielded 1,066,719 raw reads (551,935 reads from pre-parasitic stages library and 514,784 reads from post-parasitic stages library), for a total 520,863,572 bp, with an average length of 488 bp (Table 1). The comparable sequencing depth, reads length and quality (Fig. 2) allowed combined assembly of pre-parasitic and post-parasitic libraries for complete life cycle (Table 2). The final assembly resulted in total 82,212 expressed sequence tags (ESTs) consisting of 10,811 aligned contigs with an average length of 1442 bp and N50 of 1754 bp and 71,401 singletons with an average length of 430 bp (Table 2). From these contigs and singletons, we obtained 10,738 and 63,601 putative operons, respectively. Our pyrosequencing of complete life cycles of Chinese *H. avenae* produced both longer total transcripts and average ESTs in length, compared to that of the report from Kumar et al., which sequenced the two development stages (juvenile 2 and adult female) of Indian *H. avenae* (Tables 1 and 2).

**Table 1:** Summary of *H. avenae* transcriptomes sequencing from two independent studies

*H. avenae* transcriptome sequencing	This paper^a^			Kumar et al. [20]^b^	
Method	Roche FLX+			Illumina GAIIx	
Sample life stages	Pre-parasitic	Post-parasitic	Whole life stages (pre- and post-)	Hatched Juvenile 2	Feeding female
Num. of reads/PE-reads	551,935	514,784	1,066,719	46,114,413	37,125,131
Total length (Mb)	272	249	521	8795	7081
Average read length (bp)	492	483	488	2×100	2×100

^a^ This paper sequenced two mixed-stages transcriptomes of a population of *H. avenae* in China. The pre-parasitic mixed-stages included egg, juvenile 1 and hatched juvenile 2, while the post-parasitic mixed-stages included parasitic juvenile 2, juvenile 3, juvenile 4, adult female and male. The whole life stages data consisted of sequencing reads from both pre-parasitic and post-parasitic mixed-stages

^b^ Kumar et al. [20] presented original transcriptomes sequencing for two life stages of a population of *H. avenae* in India

**Table 2:** Assembly of *H. avenae* transcriptomes from two independent studies

*H. avenae* transcriptome assembled	This paper	Kumar et al. [20]
Num. of total reads	1,066,481	144,868,609
Num. of aligned reads	933,304	78,113,600
Num. of contigs	10,811	27,765
Length range of contigs (bp)	51–13,668	101–9075
Mean length of contigs (bp)	1442	682
N50 of contigs (bp)	1754	1028
Num. of singletons^a^	71,401	–
Length range of singletons (bp)	50–1248	–
Average length of singletons (bp)	430	–
Total transcripts length (Mb)	19.31	18.93

^a^ Singletons represent the reads that cannot be assembled in contigs

General annotation showed that 71.1% of contigs and 34.3% of singletons had homologous proteins from non-redundant GenBank proteins (NR) or Swiss-Prot/TrEMBL databases (BLASTX e-value <1e^−6^, Similarity >50%). Only 25,657 (30.9%) ESTs were successfully annotated by Gene Ontology (GO), of which 5816 (53.8%) contigs hit 21,633 GO terms while 19,841 (27.8%) singletons hit 129,349 GO terms as shown in Additional file 1: Figure S1a. Notably, 24,146 operons had KEGG orthologs (KO), of which 3338 were drawn on KEGG pathway maps (Additional file 1: Figure S1b). Among the 6803 contigs operons with KO, 1853 were involved in 287 biological pathways. Our subsequent analyses on pathway enrichment of significant biological categories were based on these 1853 contigs.

### Phylogeny and ortholog analyses

**Fig. 3:**
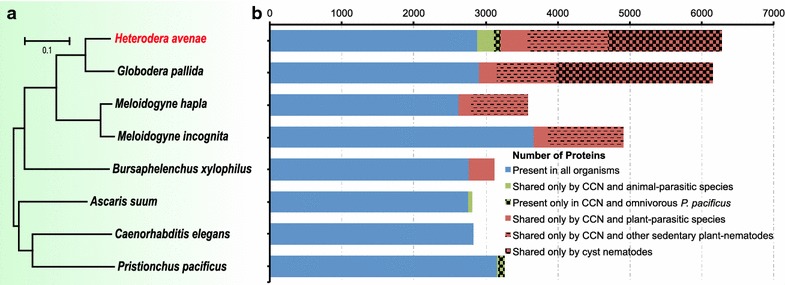
Phylogeny and ortholog distribution of 8 nematodes with diverse life styles. **a** A genome-based phylogentic tree generated by 703 single ortholog families. *Scale* (0.1) represents the number of amino acid changes per site. **b** Distribution of orthologous identified in *H. avenae* and other 7 selected nematodes with published genomic data

The phylogeny and ortholog analyses were carried out by comparing 74,339 operons of *H. avenae* with other 7 published nematode genomes (*Globodera pallida* [26], *Meloidogyne incognita* [27], *Meloidogyne hapla* [28], *Bursaphelenchus xylophilus* [29], *Caenorhabditis elegans* [30], *Pristionchus pacificus* [31] and *Ascaris suum* [32]). This resulted in the identification of 12,703 orthologous families, of which 703 single-copy ortholog families were used to draw the phylogeny tree (Fig. 3a). Representing the varied life styles of these nematodes, the tree demonstrated a clear separation of plant parasitic nematodes (*H. avenae*, *G. pallida, M. incognita*, *M. hapla* and *B. xylophilus*), animal parasitic nematode (*A. suum*) and bacterivorous free-living nematodes (*C. elegans* and *P. pacificus*). While *B. xylophilus* represented migratory nematode, cyst nematodes (*H. avenae*, *G. pallida*) and root-knot nematodes (*M. incognita*, *M. hapla*) represented nematodes of sedentary endoparasites (Fig. 3a). As a satellite model of bacterivorous *C. elegans,* omnivorous *P. pacificus* can feed on not only various bacteria, but also fungi and even other nematodes [33]. Taking together, the relative proximity in the phylogeny tree well recapitulates the similarities of life styles among these nematodes.

The 11,345 operons from *H. avenae*, which belonged to 8086 orthologous families, were subjected to categorization by the distribution of orthologous among 8 nematodes. We subdivided these orthologous families into 6 groups (Fig. 3b). There were 2248 families, including 2879 *H. avenae* orthologous, shared by all the above 8 nematodes. Notably, 3070 orthologous specifically shared by *H. avenae* and other 5 plant-parasitic nematodes, among which 2699 only shared by sedentary nematodes and 1575 uniquely in cyst nematodes. Interestingly, 92 orthologous shared by *H. avenae* and omnivorous *P. pacificus*, but not by other phytonematodes, were also found in animal parasitic nematode *A. suum*. For a detailed insight on the comparisons between *H. avenae* and other nematodes, we performed GO enrichment analysis of the common *H. avenae* orthologous (Additional file 2: Figure S2).

As a premier biological and genetic model, *C. elegans* provides a critical reference for the investigation of biological pathways shared by nematodes [30]. The *H. avenae* orthologous that are corresponding to *C. elegans* genes involved in RNAi pathway (Additional file 3: Table S1a), immune response (Additional file 3: Table S1b), diapause pathway (Additional file 3: Table S1c), and neurotransmitter biogenesis pathways (Additional file 3: Table S1d) were extracted from 12,703 orthologous families and compared among other nematodes. These comparative analyses on orthologous in these biological pathways facilitate further characterization of *H. avenae*, as well as the understanding of molecular mechanisms involved in the nematodes-plant host interactions, thus assist the identification of new targets and development of novel strategies for control of *H. avenae*.

**Fig. 4:**
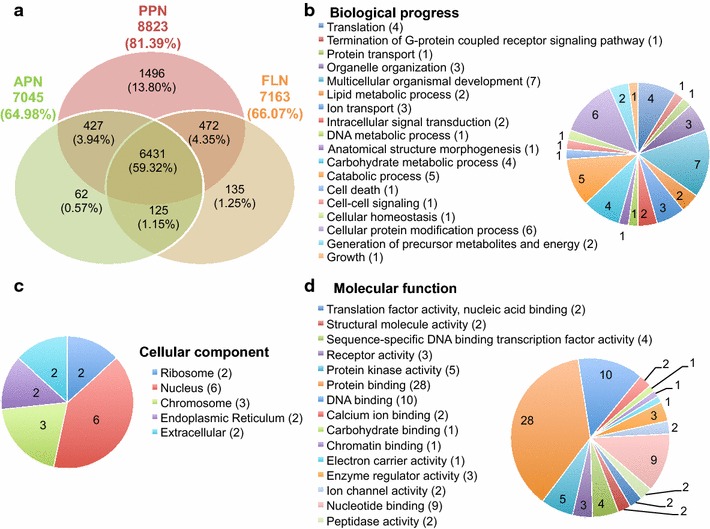
Summary and classification of ESTs in *H. avenae* transcriptome. **a** The contigs identified in *H. avenae* were compared with known ESTs from nematodes with three distinct life styles by Venn diagram. *PPN* plant parasitic nematodes, *APN* animal parasitic nematodes, *FLN* free-living nematodes. Functional classification of *H. avenae*-specific transcripts using Gene Ontology (GO) terms in the categories of biological progress (**b**), cellular component (**c**) and molecular function (**d**)

In order to find out unique genes in *H. avenae*, 10,811 contigs were compared with all the available 1,331,401 EST sequences of nematoda from NCBI and Nembase 4 (Additional file 4: Table S2) by reciprocal tblastx (cutoff score 50). As a result, 9145 contigs (84.6%) from *H. avenae* showed significant sequence similarity with other nematodes ESTs and were classified into three life styles in the venn diagram (Fig. 4a). The remaining 1666 contigs (15.4%) without obvious hit were considered as unique genes in *H. avenae*. Most of these unique ESTs did not have much annotation, and only 74 of them had GO assigns involved in biological progress (Fig. 4b), cellular component (Fig. 4c) and molecular function (Fig. 4d).

### Differential expression analyses between pre- and post-parasitic stages

In order to obtain the differential expression profiling between the pre- and post-parasitic stages, we calculated gene expression from the assembled reads of two libraries, using 10,811 assemble contigs of *H. avenae* full life cycle as reference. The pre-parasitic library, consisting of 10,176 contigs, exhibited similar expression abundance and distribution profiling with the post-parasitic library, which was composed of 9510 contigs (Additional file 5: Figure S3a, b). There were 8877 common contigs and 3378 significantly differentially expressed contigs (FDR < 0.001, |log_2_(fold change)| > 1) between these two libraries, of which 1779 contigs were highly expressed in pre-parasitic stages and 1598 contigs in post-parasitic stages, calculated by DEGseq with MA-plot-based method (Additional file 5: Figure S3c).

**Fig. 5:**
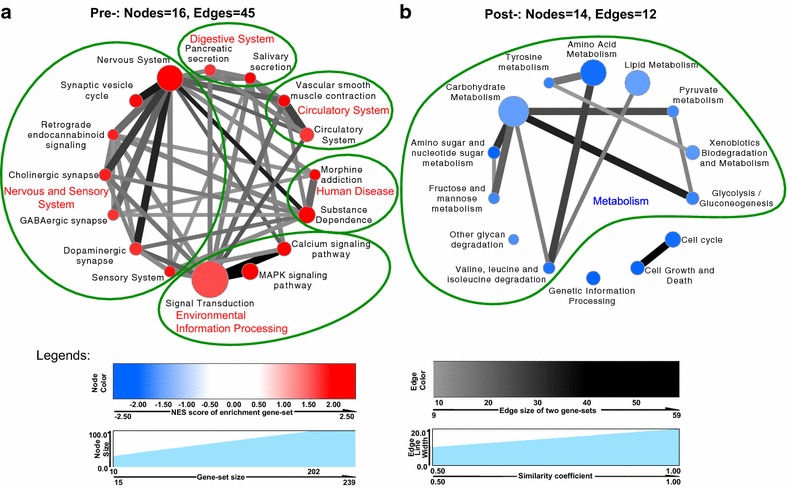
Crosstalks of significantly enriched KEGG pathways before and after parasitism in *H. avenae*. Crosstalks of significantly enriched KEGG pathways from *H. avenae* at pre-parasitic (**a**, *red nodes*) and post-parasitic (**b**, *blue nodes*) stages. Node, a KEGG pathway gene-set, and the node sizes positively correlate with the number of genes in the pathway; the darker the color is, the more significantly the gene-sets are enriched (*P* < 0.01, FDR < 0.25). Edge, the relevance of two gene-sets, and the more relevant (similarity >0.5), the more genes shared by two gene-sets; the darkness and thickness of lines positively correlate with amount of genes and similarity of two gene-sets, respectively. The normalized enrichment score (NES) is the primary statistic for evaluating gene set enrichment by GSEA

Furthermore, we generated 330 biologic pathways gene-sets from 1853 *H. avenae* contigs assigned by KEGG pathway, of which 569 contigs exhibited significantly differential expression before or after parasitism. To discern telltale biological themes, the 569 differential expressed contigs were ranked from the expression datasets and examined for the statistically significant, concordant differences by Gene Set Enrichment Analysis (GSEA) with a weighted resampling based pathway enrichment test. As demonstrated in the layout visualization by Enrichment Map, there were 30 crosstalk pathways significantly enriched at the cut off level of *p* values <0.01, FDR < 0.25, similarity >0.5, of which 16 pathways were actively involved in pre-parasitic stages and 14 pathways in post-parasitic stages (Fig. 5). For pre-parasitic stages, 16 nodes linked by 45 edges indicated the crosstalk events. The grouped big clique represented the active pathways in digestive system, circulatory system, human disease, nervous/sensory system and environmental information processing (Fig. 5a). However, for *H. avenae* at the post-parasitic stages, significantly enriched biological processes were focused on metabolism, genetic information processing, cell cycle and cell growth and death, which were denoted by three clique-groups consisting of 14 nodes linked by 12 edges (Fig. 5b). The heat map depicting the expression profiles of crosstalk genes relevant to these pathways is shown in Additional file 6: Figure S4. The differentially expressed genes in each node and the normalized quantification were listed in Additional file 7: Table S3.

### High expression of neuropeptides at pre-parasitic stages

**Fig. 6:**
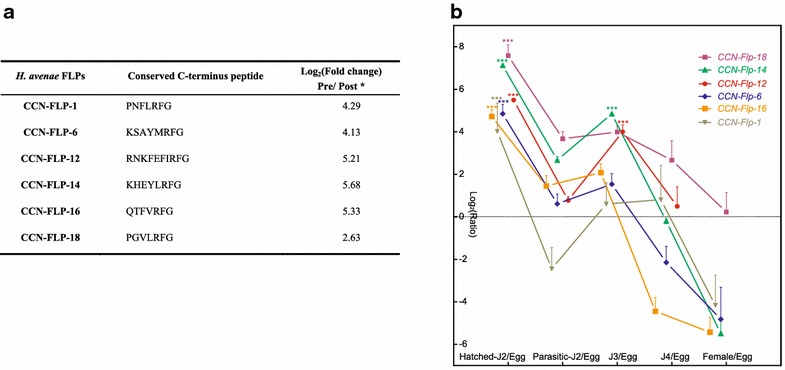
Differential expressions of FMRFamide-like peptides before and after parasitism in *H. avenae*. **a** Comparison of expression levels of indicated FLPs at pre-parasitic and post-parasitic stages by analyzing transcriptome-sequencing data. * Log_2_(fold change) pre/post, normalized Log_2_(pre-parasitic RPKM/post-parasitic RPKM), calculated by DEGseq. **b** Real-time PCR results for the expression of indicated FLPs genes in five different developmental stages. The FLPs expression levels in *H. avenae* at hatched-J2, parasitic-J2, J3, J4 and female adult stages were normalized to that of egg stage. Statistic significance was determined by one way analysis of variance (ANOVA) test. ***p < 0.001

Acting as neuromodulators and primary transmitters, neuropeptides have a diverse role in the development and function of nervous system in nematodes [34–36]. The main role of neuropeptides is as modulators of synaptic activity in a range of processes including sensory perception, locomotion, development, egg-laying and dauer formation [26]. FMRFamide-like peptides (FLPs), named from the sequence Phe-Met-Arg-Phe-NH2, are essential neuropeptides with diverse physiological functions [37]. Interestingly, we found a significant decline of genes coding for FLPs in our *H. avenae* transcriptome data after parasitism (Fig. 6a). These neuropeptides were identified as CCN-FLP-1, CCN-FLP-6, CCN-FLP-12, CCN-FLP-14, CCN-FLP-16 and CCN-FLP-18. Consistent with this notion, the quantitative PCR analysis further confirmed that all of these FLPs exhibited remarkably highest expression at the hatched-J2 stage (Fig. 6b). Although a peak expression was observed at J3 stage for each FLP, the expression level at J3 stage was substantially lower than that at pre-J2 stage. Notably, even lower expression of each FLP was found at the J4 and adult female stages. Our results support the notion of vital roles of enriched FLPs genes for nervous system before invasion, providing highly valued targets for pharmacological interference prior to the parasitism of *H. avenae*.

### Potential effectors of *H. avenae*

Decades of research demonstrates that numerous proteinaceous effectors are synthesized in the esophageal gland cells with N-terminal signal peptides, which direct the effectors to the classic secretory pathway [7]. However, effectors without signal peptides are also identified from the extracellular culture by protein mass spectrometry [11]. We identified total 4229 putative secretory proteins, from our *H. avenae* transcriptomes, as the proposed effectors repertory. This repertory consisted of 3985 signal peptide-marked proteins and 244 operons with secretory homologues. Notably, 59 published phytonematodes key effectors (without CAZymes) that had homologues in *H. avenae* were listed in Additional file 8: Table S4a.

Carbohydrate-active enzymes (CAZymes) secreted by nematodes to modify plant cell walls, are essentially considered as critical factors for successful parasitism of plant host [7]. We identified 261 *H. avenae* operons with 270 CAZymes modules classified into 87 families by searching dbCAN database. The *H. avenae* ESTs coding for CAZy enzymes with homologies to that of other phytonematodes were listed in Additional file 8: Table S4b, and interestingly, the majority had an expression bias at pre-parasitic stages. This notion was supported by the RT-PCR analysis of cellulases (*Ha*-*eng*-*2* and *Ha*-*eng*-*3*) reported by Long et al. [38]. These two CAZymes had the most transcriptions in the hatched-J2s, medium transcriptions in the early parasitic-J2s, and almost undetectable transcriptions at the late parasitic stages of *H. avenae* [38]. It is possible that the accumulation and stock of mRNA for these CAZymes might be a prerequisite for initial infection of host plant, because a large number of templates for protein translations are urgently needed upon invading the host tissue.

RxLR effectors facilitate the transportation of cytoplasm proteins from malaria parasite to the host and assist suppression of the defense of plant-host triggered by microbe pathogens [39, 40]. They are characterized by a highly conserved region defined by the invariant RxLR motif sequence (R, Arg; L, Leu; x, any residue) within the N-terminal 30–60 amino acids after secretory signal peptides [41]. Surprisingly, we also found 61 predicted secretory peptides with RxLR motif within the N-terminal 100 amino acids in our *H. avenae* transcriptome as putative RxLR effectors. Among them, 10 candidates had RxLR-[E/D/Q] motif, which was considered as host-targeting motif aiding effector export from malaria parasite [39, 42]. We listed 15 putative RxLR/RxLR-[E/D/Q] effectors with homologues in NR database (blastp, e-value <1e^−5^) (Table 3). Our results imply the possibility of existence for pioneer RxLR effectors in *H. avenae*.

**Table 3:** Putative transcripts with RxLR motif in *H. avenae* with NR hits (Blastp, E value <1e^−5^)

CCN protein	SP site^a^	RxLR		Hit accession	Description [species]	E value	Ident (%)	Expression bias
		Start	Motif					
ISOTIG15357	27	31	RqLR	KHN74319	Chondroitin proteoglycan 2 [*Toxocara canis*]	4.00E−11	30	Post
ISOTIG17027	26	39	RsLR	KHN88475	Hypothetical protein Tcan_06414 [*Toxocara canis*]	2.00E−104	69	–
HOWVQCB02JEWZI_10	22	48	RlLR	KHN81668	Hypothetical protein Tcan_03447 [*Toxocara canis*]	3.00E−13	36	Pre*
ISOTIG16444	28	53	RlLRQ	CTP81713	BMA-SOL-1 [*Brugia malayi*]	7.00E−53	46	–
HOWVQCB02JXEXX_14	32	53	RmLR	CCA67693	Hypothetical protein PIIN_01520 [*Serendipita indica DSM 11827*]	2.00E−24	86	Pre*
ISOTIG15922	24	58	RlLR	AEH42093	Cysteine proteinase 6 [*Haemonchus contortus*]	1.00E−38	37	Pre
ISOTIG07167	20	59	RlLRD	CDW60295	SPRY domain containing protein [*Trichuris trichiura*]	3.00E−06	35	–
HOWVQCB02FXQYR_12	33	60	RlLR	KHN81668	Hypothetical protein Tcan_03447 [*Toxocara canis*]	4.00E−13	34	Pre*
ISOTIG15903	29	63	RaLR	XP_003145427	Leukocyte cell-derived chemotaxin 2 [*Loa loa*]	3.00E−97	46	–
ISOTIG17370	23	65	RvLR	AAG21334	Hypothetical esophageal gland cell secretory protein 4 [*Heterodera glycines*]	3.00E−76	47	Post
HPF5SIP01EI8EZ_11	40	69	RmLR	XP_001895511	Histone H2A [*Brugia malayi*]	8.00E−56	76	Post*
HPF5SIP01BKTZ3_5	36	69	RiLR	XP_002677860	Hypothetical protein NAEGRDRAFT_79489 [*Naegleria gruberi*]	1.00E−34	49	Post*
ISOTIG10016	28	74	RkLR	KHN78406	Thioredoxin domain-containing protein C06A6.5 [*Toxocara canis*]	0	64	Post
ISOTIG09138	18	78	RmLRE	EGT56409	CBN-LET-268 protein [*Caenorhabditis brenneri*]	3.00E−122	45	Post
HO9SJLY04I1AHW_9	39	89	RyLR	KHN81115	La-related protein [*Toxocara canis*]	3.00E−26	50	Pre*

‘Pre*/post*’ means coding by singleton in pre-/post-parasitic library. The singleton might be the gene with low expression and was sequenced only once

^a^ SP site means signal peptide cleavage site. NR means non-redundant GenBank proteins database. ‘Pre/post’ means significantly high expression in pre-/post-parasitic stage

**Fig. 7:**
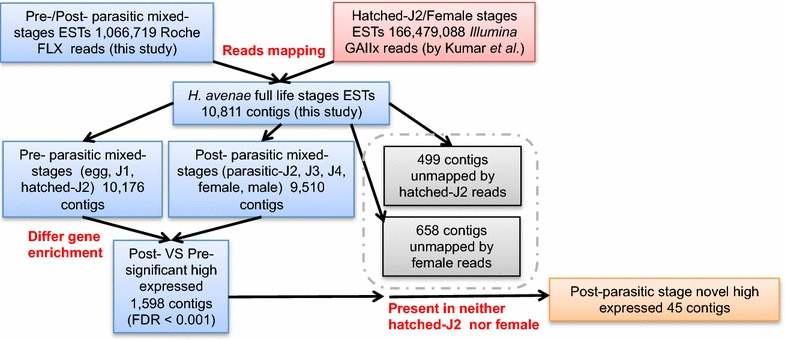
The schematic representation of the screening pipeline for highly expressed parasitism-specific genes in *H. avenae*. The gene contigs significantly enriched in post-parasitic stages [FDR < 0.001, Normalized log_2_(fold change) >1] but without expression in neither hatched-J2 nor adult female stages are considered potentially parasitism-specific genes

Effectors secreted during parasitism play vital roles in establishing and maintaining feeding site of a permanent source of nutrients, as well as in response to immune defense of plant hosts [7, 9, 43]. Based on the available transcriptome sequencing data of *H. avenae*, we designed a strategy to mine the potential effectors highly expressed during parasitism (Fig. 7). The raw sequencing reads of 4 libraries from differed samples, including *H. avenae* at the hatched-J2 stage [20], adult female stage [20], pre-parasitic mixed-stages (egg, J1, hatched-J2) and parasitic mixed-stages (parasitic-J2, J3, J4, female, male) were mapped to the reference transcripts of whole life *H. avenae* (derived from our 10,811 configs), respectively. The statistic numbers of mapped reads revealed the expression of transcripts from each sample. After strict screening, the 45 transcripts that significantly enriched in post-parasitic mixed-stages (FDR < 0.001) but displayed no read mapped from neither hatched-J2 nor adult female stages were considered as potentially key factors at the earlier stage of parasitism phase. Among these 45 transcripts, there were 3 transcripts that did not have operon, and 13 transcripts had operons with predicted secretory signal peptides. Markedly, we found 16 transcripts that possess obvious homologous by searching NR database (blastx, e-value <1e^−5^) (Table 4). Interestingly, 3 *H. avenae* transcripts that share strong sequence and structural resemblances with *Toxocara canis* ANT-5/ANT-34 and flavivirus NS2/NS5 were not identified in any other plant nematodes.

**Table 4:** Transcripts specifically highly expressed at *H. avenae* parasitic stages (except adult female) with NR hits (Blastx, E value <1e^−5^)

CCN contig	Log_2_(FC)	Q value	Hit accession	Description [species]	E value	Ident (%)
isotig13402	6.89	3.35E−134	YP_009029999	NS5-like protein [*Jingmen tick virus*]	3.00E−91	31
isotig13435	6.53	1.72E−127	ACF19855	ANT-34 [*Toxocara canis*]	6.00E−48	32
isotig14470	5.78	1.09E−07	WP_014934453	50S ribosomal protein L25/general stress protein Ctc [*Cardinium endosymbiont of Encarsia pergandiella*]	1.00E−86	74
isotig12660	5.12	2.24E−05	WP_068700363	Glycoside hydrolase [*Paenibacillus yonginensis*]	1.00E−26	45
isotig15541	4.98	1.88E−08	EMG48930	Hypothetical protein G210_0425 [*Candida maltosa Xu316*]	0.003	29
isotig17002	4.94	3.26E−08	WP_011271320	Heat-shock protein C2 [*Rickettsia felis*]	1.00E−41	55
isotig19536	4.94	6.67E−05	WP_034577564	Preprotein translocase subunit SecE [*Cardinium endosymbiont of Bemisia tabaci*]	8.00E−25	77
isotig19691	4.84	1.15E−04	XP_002672706	Predicted protein [*Naegleria gruberi*]	5.00E−33	48
isotig15266^a^	4.73	1.99E−04	XP_015393075	PREDICTED: cuticle protein 12.5-like [*Panthera tigris altaica*]	1.00E−16	41
isotig18086	4.49	6.12E−04	ACF19853	ANT-5 [*Toxocara canis*]	1.00E−38	51
isotig20036	4.49	6.12E−04	KNA02486	Hypothetical protein PVNG_03779 [*Plasmodium vivax North Korean*]	5.00E−12	77
isotig14974	3.61	7.60E−04	WP_034577458	Transcription termination/antitermination protein NusA [*Cardinium endosymbiont of Bemisia tabaci*]	0	76
isotig16565	2.92	2.52E−07	KFH65214	Hypothetical protein MVEG_08695 [*Mortierella verticillata NRRL 6337*]	1.00E−22	45
isotig16605	2.73	2.34E−05	WP_014934894	50S ribosomal protein L20 [*Cardinium endosymbiont of Encarsia pergandiella*]	1.00E−55	87
isotig14490	2.54	5.90E−07	XP_005705008	DNA-directed RNA polymerase subunit alpha [*Galdieria sulphuraria*]	4.00E−58	45
isotig16931	1.72	5.88E−04	WP_014934892	Translation initiation factor IF-3 [*Cardinium endosymbiont of Encarsia pergandiella*]	4.00E−82	77

^a^ With signal peptides. Log_2_ (FC) = normalized Log_2_(post-parasitic RPKM/pre-parasitic RPKM). Both Log_2_ (FC) and Q value threshold (Storey et al. [44]) were calculated by DEGseq. NR means non-redundant GenBank proteins database

## Discussion

Without genomic reference, we chose the relatively more costing platform of 454 FLX+/Roche to sequence the transcriptomes of *H. avenae* at pre-parasitic mix-stages (including eggs, J1 and hatched-J2) and post-parasitic mix-stages (including parasitic-J2, J3, J4, and adults) (Table 1). Our de novo assembly of 82,212 expressed sequence tags (ESTs) that covered the entire life cycle of this parasite was benefited from the long reads of combined libraries. Among these ESTs, 10811 contigs had notable lengths with N50 of 1754 bp, while 71,401 singletons had an average length of 430 bp (Table 2). Besides the transcriptomes of two life stages (hatched-J2 and feeding female) sequenced by Kumar et al. and the parasitism genes reported by Zheng et al. during incompatible invasion [20, 25], our data substantially enriched the genetic information of the cereal pathogen *H. avenae*.

Orthologs are genes in different species that evolved from a common ancestral gene by speciation, normally retaining the same function in the course of evolution [45]. For reliable prediction of gene function, we identified and compared orthologs from the putative translated products of *H. avenae* trancriptomes and other completely sequenced genomes of 7 nematodes, which are representative species with different life styles. As expected, there are great correlations between the similarity of life styles and the proximity of compared nematodes in phylogenetic tree generated from single copy ortholog families (Fig. 3). The closest species to *H. avenae* is *G. pallida*, which agrees with the report from Kumar et al., because they belong to cyst genus and have the most similar parasitic life style. In addition, our GO enrichment analyses show that the organonitrogen compound metabolic process is unique in the cyst nematodes (Additional file 2: Figure S2D). Moreover, the GO terms on single-organism cellular process and neurological system process are shared only by sedentary nematodes (Additional file 2: Figure S2c). Like other sedentary phytonematodes, *H. avenae* has evolved a special sensorial processing system that is distinguished from migratory parasites during obligate sedentary parasitism. Surprisingly, three *H. avenae* ESTs have been identified with sequence similarity to *Ants*, which are only shared by animal parasitic nematode and *H. avenae* (Fig. 4a). The potential role of *Ants* as novel functional effectors is discussed below.

Currently, RNAi technology has become one of the predominant tools for studying gene function in phytonematode, as well as an effective anti-parasite strategy. Nevertheless, some indispensable factors for the RNAi pathway in *C. elegans* (Additional file 3: Table S1a), such as *sid*-*1* [46, 47] and *sid*-*2* [48], which are required for the uptake of dsRNA in the intestine, are not identified in *H. avenae* and any other phytonematodes. In fact, repeated reports of successful silencing of target genes by feeding dsRNA using socking stimulant or host-derived delivery [49, 50] imply that phytonematode like *H. avenae* might have alternative signaling pathway for up taking of dsRNA in the intestine. The variance on dsRNA processing between *H. avenae* and *C. elegans* remains a subject for further investigation, and the better understanding will definitely illuminate new ideas for the control of *H. avenae*.

Like *C. elegans* [51] and *G. pallida* [26], *H. avenae* relies tightly on innate immunity to defend the infection and infiltration of different microbial pathogens. As compared with other sedentary nematodes, we found that the overwhelming majority of immune signaling pathways appeared to be highly conserved between *H. avenae* and *C. elegans*, except *ikb*-*1* in the toll-signaling pathway (Additional file 3: Table S1b). The relative conservation of innate immune defense system in *H. avenae* and *C. elegans* highlights the potentials of disrupting the immune system to increase the susceptibility of *H. avenae* to microbial infection for biocontrol.

Diapause under hot dry conditions in summer is one of the unique habits of *H. avenae.* However, the facultative diapause ability of the populations of Chinese *H. avenae* is distinct from that of the populations in Mediterranean climates. Their dormancy is also promoted after chilling in winter, which ensures the emergence of infective juveniles upon temperature rising in spring [52–54]. This facultative diapause of *H. avenae* in China, regulated by different thermal conditions, is the same case in the laboratory [55]. Thus, the characterization of the signaling pathways that control the developmental decision to enter and leave the dauer stage is essential to manipulate the life cycle of *H. avenae* for control. In *C. elegan*, many mutants with high temperature induction of dauer (Hid) phenotype have defects in synaptic transmission, highlighting the neuroendocrine nature of dauer regulation [56–58]. Although the sensory of Hid couples downstream to insulin-like and TGFβ-like signaling pathways in *C. elegan* [57, 59, 60], we did not identify these two intact dauer regulatory pathways from both *H. avenae* and *G. pallida,* Only Guanylyl cyclase pathway was conserved in both *H. avenae* and *G. pallida* with reference to the genome of *C. elegan*. 8-bromo-cGMP (8Br-cGMP) functions in this pathway to regulate dauer arrest in both *C. elegans* and *G. pallida*. While 8Br-cGMP rescued the phenotype of constitutive dauer in *daf*-*11* mutants [61], study in *G. pallida* also demonstrated that 8Br-cGMP facilitated the release of diapause by activation of hatching [62]. Due to the functional conservation of guanylyl cyclase pathway between *C. elegans* and cyst nematodes (Additional file 3: Table S1c), it is deducible that manipulation of this pathway might be effective for controlling the hatching of *H. avenae*.

To provide novel insights into the differences of *H. avenae* at stages before and after parasitism, 3378 significantly differentially expressed contigs were gained from the comparison of transcriptomes. The divergence of enrichment KEGG pathways of these differential expression genes indeed reflects distinct demands for survival before and after parasitism. In the pre-parasitic stages, the active highly socialized pathways, which consisted of environmental information processing system, nervous system, digestive system, circulatory system and human disease (Fig. 5a), benefit the adaptability and motion of *H. avenae* in soil and facilitate the perception of complex ambient stresses and invasion of host plant. From the above crosstalk pathways, nervous system probably play a signaling or integrative role in the pre-parasitism activities of *H. avenae*, such as the defense against the microbial pathogens [63] in soil, temperature induction of dauer [60] and seeking for the host plant. After successful parasitism, metabolism related pathways, including amino acid metabolism, biosynthesis of other secondary metabolites, carbohydrate metabolism and lipid metabolism, were predominant to coordinate the production of endocrine hormone, cellular proliferation and differentiation, as well as cell apoptosis and death (Fig. 5b). Probably the secretory molecules in these pathways that can be delivered into host plant cell and further regulate host plant-nematode interaction are putative effectors, which essentially benefit the initiation and sustaining of parasitic life for *H. avenae*.

As demonstrated previously, there is significant neurological system in sedentary nematodes (Additional file 2: Figure S2c) and an obvious bias of active biosynthesis of neurotransmitters towards the pre-parasitic stages of *H. avenae* (Fig. 5a). Substantially, neurotransmitters contribute to the complex and subtle behaviors of nematodes, such as recognition of hosts, defense to pathogens and response to environmental stresses [26, 64]. Thus, the better characterization of the synthesis, transportation and metabolism of neurotransmitters may help exploiting particular targets for rational chemical control methods. As compared to *G. pallida*, another well characterized cyst nematode, *H. avenae* had the same genes profile in the majority of signaling pathways like acetylcholine and dopamine (Additional file 3: Table S1d). However, serotonin biosynthetic enzyme coding gene *tph*-*1* was absent in *H. avenae*. It is worth noting that the octopamine, glutamate, GABA signalling pathways were highly conserved in all the nematodes, which implies the universality of existed and novel strategies targeting these neurotransmitter pathways. For instance, octopamine is used to stimulate ingestion of dsRNA in invitro RNAi experiments by increasing the pharyngeal pumping of plant nematodes [65]. On the other hand, since octopamine displays roles in inhibition of egg laying in *C. elegans*, investigators have adopted octopamine as an effect approach in the control of phytonematodes [66, 67]. Our greater understanding of the available target molecules will considerably help in the rational design of new biological nematicide, as well as in the development of next-generation chemical nematicide with minimal adverse effects.

In addition, neuropeptides may also function as primary neurotransmitters. The FMRFamide-like peptides (FLPs) are broadly conserved neuropeptides in nematodes, while the profiling of FLPs varies among phytonematode and other nematodes in terms of structure, copy, expression location and developmental expression pattern [37, 68]. For example, N-terminal variations of FLP-12 exist among the animal parasites, plant parasites *Heteroderidae* spp. and *Meloidogyne* species [37]. The FLP-6 expression was observed in the circum pharyngeal nerve ring (main response pharyngeal pumping) of potato cyst nematode of *G. pallida*, whereas FLP-6 in bacterivorous *C. elegans* was reported solely in the ASE head neurons [68]. Paul McVeigh et al. reported that there is a general expression bias of FLPs towards Clade V species (such as *C. elegans*, *P. Pacificus*), appearing to be over represented at L3 stage juveniles and female worms, according to the analysis of EST database sequences [37]. However, our investigation demonstrated that *H. avenae*, as a Clade VI plant parasite, had highest expression of a series of FLPs at hatched J2 stage before parasitism in plant host (Fig. 6). These distinctions of FLPs among nematodes indicate that the special roles of FLPs are inextricably linked to parasite motor and sensory function, particularly in plant nematodes.

Disruption of parasite FLP signals may be considered as an approach for developing new specific nematocides. Since our identified FLPs (CCN-FLP-1, CCN-FLP-6, CCN-FLP-12, CCN-FLP-14, CCN-FLP-16 and CCN-FLP-18) in *H. avenae* had major expression peak at hatched-J2 and minor expression peak at post-parasitic J3 stages (Fig. 6b), they have obvious potentials as targets for parasite control on both ecto- and endoparasitic phases. In deed, significant reduction of infection and multiplication of *M. incognita* was observed in the genetically engineered tobaccos using plant-mediated RNAi of nematode FLP-14 or FLP-18 [69]. We still know relatively little about the downstream signaling processes through which the effects of these FLPs are exerted. However, on the other hand, the signaling molecules and receptors involved in these pathways could also be the targets for pharmacological interference and the control of phytonematodes.

Numbers of putative effectors were mined from *H. avenae* transcriptomes. The key genes identical from phytonematodes reported effectors can be categorized into the following groups [9]: (1) effectors for invasion, extension and degradation of host tissues. This group is dominated by Cazymes, such as cellulase, pectate lyase, calreticulin, expansin; (2) effectors for regulating plant cell division and differentiation to maintain the integrity of syncytium for the feeding site, such as CLE-like proteins; (3) effectors that protect nematodes from anti-pathogen compounds in host defenses, such as fatty acid and retinol binding protein, peroxiredoxin; (4) effectors that target plant signalling pathways and suppress host defenses, such as annexin, venom allergen like protein, transthyretin-like protein, ubiquitin extension proteins; (5) effectors required for feeding efficiency, such as cathepsin; (6) putative effectors with unknown function, such as 14-3-3b protein and some putative secretory proteins identified in esophageal gland cells (Additional file 8: Table S4).

The RxLR-[E/D/Q] motif, serving as an export signal at the N-terminus of cargo proteins, was firstly explored from the virulence proteins of *Plasmodium falciparum* [39]. While a family of host-translocated effectors with same structure, produced by oomycete pathogens, are termed RxLR effectors and may manipulate host physiological and biochemical events in host cells [41]. Previous studies reveal that RxLR effectors of oomycete may suppress callose deposition and cell death triggered by multiple elicitors, impair plant immunity and facilitate bacterial colonization [40]. From these results, the major function of the secretory RxLR-[E/D/Q] motif bearing proteins is concluded to be host-translocated effectors and suppress the host immunity. Notably, a SPRY domain containing protein (ISOTIG07167) was identified from our exploiting of the pioneer effectors with RxLR-[E/D/Q] in *H. avenae*. The secreted SPRY domain (SPRYSEC) family proteins from the potato cyst nematode *G. rostochiensis*, were reported to suppress plant hypersensitive response (HR) induced by effector-triggered immunity [69, 70]. The pro-apoptotic protein BAX can also trigger plant programmed cell death (BT-PCD), physiologically resembling what associated with plant HR [71]. The ability to suppress defense-associated BT-PCD has proven to be a valuable initial screening method for microbial plant-pathogen effectors [72, 73]. Interestingly, ISOTIG17370, as a candidate without pentameric motif RxLR-[E/D/Q] but as a homolog of esophageal gland cell secretory protein 4 in *Heterodera glycines* (a soybean cyst nematode) [15], could suppress BT-PCD in our preliminary experiments. It is possible that some RxLR effectors, even without RxLR-[E/D/Q] core, might be still actively involved in inhibition of plant immunity (like oomycete), although the underlying mechanisms on how they enter host are still unknown.

From the exploring by combinational analyses of our and another reported transriptomes for *H. avenae*, we captured 16 highly expressed candidate key genes that putatively play essential roles during parasitism (Fig. 7). Five of these genes were suggested to be regulators and participators of RNA transcription, such as translation initiation factor IF-3, transcription termination/anti-termination protein NusA, DNA-directed RNA polymerase and ribosomal protein (Table 4). ANTs (abundant novel transcripts) are one group of highly enriched transcripts initially identified from the infective stage of *Toxocara canis* in nematoda [74]. It is worth noting that three putative parasitism-specific ESTs in *H. avenae* share sequence similarity to ANT-5/ANT-34 or NS2/NS5. They were also considered as the inhibitors of transcription in both *Toxocara canis* and flaviviruses [75]. To our knowledge, genes homologous to ANTs only exist in *H. avenae* and *T. canis*, among all the nematoda with available sequencing data. ANT-5 and ANT-34 are homologous of NS2 and NS5 in flaviviruses, and share strong sequence and structural resemblances of NSP1 and NSP2 in a tick-borne virus-Jingmen tick virus [76]. ANT-5 shows distant similarity to RNA regulatory protein and RNA-dependent RNA polymerase, while the 3′UTR of ANT-34 greatly reduces reporter gene expression, inhibiting both transcription and translation [75]. Anti-ANT-34 antibody had been found in the sera of hosts with *Toxocara canis* infection via high-affinity immune assays [75]. These points to the possibility that ANTs interact with the host and play roles in the extracellular environment although they could be RNA regulatory proteins and without identifiable N-terminal secretory signal peptides. Thus, it is plausible that ANTs from *H. avenae* could also be secreted and transported into host plants to function as effectors. We did not find the expression of secretory signal peptides for 32 putative effector ESTs. This is perhaps due to the lack of intactness in N-terminus of sequenced ESTs. However, it is also possible that these effectors share a non-canonical secretory pathway, just like ANTs.

## Conclusion

Taking together, we present the transcriptomic data of *H. avenae* at pre- and post-parasitisitic stages. To our knowledge, this is the first report on *de novo* analysis of transcriptomes covering the complete life stages of Chinese *H. avenae*. The comparative analyses of transcriptomes for *H. avenae* and other nematodes facilitate the molecular characterization of *H. avenae* in parasitism, development, metabolism, immune defense and life style, providing guidance of multiple control approaches. Our data on in-depth mining of the potential pioneer effectors should be valuable resources not only for better understanding of the unique biology of *H. avenae*, but also for further developing possible targets-orientated strategies on effective control of *H. avenae*.

## Methods

### Collection and mass rearing of *H. avenae*

*Heterodera avenae* nematodes were collected from the field of Baoding city in Hebei province of China, identified by morphology and internal transcribed spacer (ITS)-ribosomal (r)DNA [77], and propagated on wheat (Aikang 58) from initial single cyst in an artificial environment. In this study, *H. avenae* of pre-parasitic stages (Fig. 1) included embryo eggs and the first stage of juveniles (J1s) gathered from crushed newly formed cysts or cysts incubated at 4 °C for 45 days, and the hatched-J2s collected by hatching cysts at 15 °C. The post-parasitic stages (Fig. 1) included parasitic J2, J3, J4, and adult female and male worms were isolated from the infected wheat roots digested by 6% cellulose, and distinguished under dissecting microscope as described previously [78]. Both pre-parasitic and post-parasitic *H. avenae* samples were frozen at −80 °C for later RNA isolation.

### RNA isolation, cDNA library preparation and sequencing

Frozen nematodes of various developmental stages were pooled and categorized to be the pre-parasitic and post-parasitic samples. The mRNA was isolated by Dynabeads mRNA Direct Kit (Invitrogen, USA) according to the manufacture’s instructions. Further purification was performed using MicroPoly(A) Purist Kit (Ambion, USA) after removal of trace amounts of DNA contamination with DNase I (Ambion, USA). NanoDrop and Agilent 2100 Bioanalyzer assessed the quantity and quality of purified mRNA, respectively. For 454 pyosequencing, the first-strand cDNA was synthesized from mRNA reverse transcription with Superscript II Reverse Transcriptase (Invitrogen, USA) and GsuI-oligo dT primer, and biotinylated at 5′ cap structure. The biotinylated first-strand cDNAs were released from mRNA/cDNA complexes by alkaline lysis and purified with Dynal M280 microbeads (Invitrogen, USA). Second strand cDNA was synthesized by Ex Taq polymerase (Takara, Japan) after adding 5′ adaptors with DNA ligase (Takara, Japan). The cDNA was purified by Ampure beads (Agencourt, USA) after removing polyA and 5′cap using Gsu I enzyme (Fermentas, USA).

Single stranded template DNA library was prepared by GS DNA Library Preparation Kit (Roche Applied Science, USA), and subsequently amplified by GS emPCR kit (Roche Applied Science, USA). The resultant two libraries of pre- and post- parasitic mix-stages cDNA pools were sequenced using Roche 454 Genome Sequencer FLX at Chinese National Human Genome Center at Shanghai, China.

### De novo transcriptome assembly and annotation

The reads of two libraries were combined and assembled using Roche official software Newbler 2.7 (454 Life sciences, Branford, CT, USA) at the default setting. The open reading frame (ORF) prediction was identified by using an in-house developed program based on ‘GetORF’ from EMBOSS [79]. To identify homologues of *H. avenae* transcripts in public databases, BLASTx searching was performed against Swiss-Prot/TrEMBL (http://www.ebi.ac.uk/) and non-redundant GenBank proteins (NR) (http://www.ncbi.nlm.nih.gov/) databases at an E value <1e^−5^. For function annotation, Gene Ontology (GO, http://www.geneontology.org/) analysis was performed with Blast2GO [80].

The KEGG pathway was constructed using the KEGG Automatic Annotation Server (KAAS) with BBH (bi-directional best hit, http://www.genome.jp/tools/kaas/) method [81].

### Phylogenetic and comparative analysis of orthologous families

Based on all-to-all blast (E value <1e^−6^) of predicted proteins from *Globodera pallida* [26], *Meloidogyne incognita* [27], *Meloidogyne hapla* [28], *Bursaphelenchus xylophilus* [29], *Caenorhabditis elegans* [30], *Pristionchus pacifics* [31] and *Ascaris suum* [32] genomes and *H. avenae* transcriptomes, orthologous families were constructed by orthAgogue [82] (default parameters) and MCL [83] (I = 1.5). The 703 single-copy ortholog families of all 8 nematodes were subjected to multiple global alignment by MUSLE [84] and trimming by trimAI [85]. The phylogenetic tree was inferred by MEGA6 [86] using neighbor-joining method with 1000 bootstraps replicates. The GO term enrichment analyses of *H. avenae* genes of different orthologous families groups were performed using ontologizer with term-to-term or parent–child-intersection approach and Bongerroni correction [87]. Genome datasets and affiliated annotations were downloaded from Wormbase WS245 (http://www.wormbase.org/). Sequences for *G. pallida* were from website (ftp://ftp.sanger.ac.uk/). The reciprocal tblastx (Score bits >50) strategy was used in the nematode ESTs comparisons. All the nematoda ESTs, consisting of 1,331,401 ESTs sequences from 45 genuses and 75 species clustered into three life styles, were from NCBI and Nembase 4 [88] databases (Additional file 4: Table S2).

### Comparison of gene homologues in critical biological pathways

The genes involved in critical biological pathways of *C. elegans*, including immune response pathway [51], diapause pathway [60], neurotransmitter synthesis, transportation and metabolism pathway [89], and RNAi pathway [90] were well defined by previously published literatures. The orthologous of genes in other nematodes were identified based on the orthAgogue results and manual confirmation through blast, largely following the methods used for analyses of *G. pallida* and *M. incognita* genome datasets [26, 27].

### Analyses of differential gene expression and KEGG pathway enrichment

For each contig of *H. avenae*, the number of reads was extracted from the raw assembly file (ACE file) corresponding to two parasitic stages and transformed into RPKM (reads per kilo bases per million reads) [80]. Differently expressed contigs were identified statistically by DEGseq package using Fisher’s exact test with MARS (MA-plot-based method with Random Sampling model) method [91]. We used “FDR ≤ 0.001 and the absolute value of log_2_(fold change) ≥1” as the threshold to judge the significance of contig expression differences.

A total of 330 pathways and 1853 contigs were collected from KEGG assigned *H. avenae* contigs. To avoid pathways defined for too specific or too general biological processes, we selected those with at least 15 and at most 500 genes, resulting in 184 valid pathways for our subsequent analysis. The normalized log_2_(fold change) value calculated by DEGseq was considered as expression data for *H. avenae* contigs. Among 1853 contigs, 569 differential expressed contigs were ranked and examined for significant over- or down- representation using the threshold-free technique of Gene Set Enrichment Analysis (GSEA) with a weighted resampling Kolmogorov–Smirnov-like statistic. The enrichment result was visualized by Enrichment Map (*P* value <0.01, FDR < 0.25 and similarity <0.5) as a plugin of Cytoscape [92]. The heatmap matrix of genes expression profile that represented the enriched crosstalk edges was drawn by Pheatmap of R package [93].

### Mining of putative candidate effectors

To gain the secretome repertory of *H. avenae*, we filtered the predicted products of operons with signal peptides lacking transmembrane domains by SignalP 4.0 [94] and TMHMM 2.0 [95], and searched the notable homologues from a collection of published secretomes of other nematodes with reciprocal blast (E value <1e^−5^, and minimal overlap >60%). The putative *H. avenae* homological effectors for published plant parasitic key genes were selected after manual validation. Genes encoding putative carbohydrate-active enzymes (CAZymes) were identified by HMM profiles searching as described previously [18]. RxLR effectors were acquired by structure scanning with ps_scan.pl of ScanProsite modular [96].

For the mining of potential effectors genes highly expressed during parasitism, the raw Illumina reads pairs of *H. avenae* transcriptomes by Kumar et al. [20]. were mapped to the assembled ESTs contigs of the whole life stages of *H. avenae* in this study by Bowtie2 [97]. Statistics of contigs without mapping reads from hatched J2 and female stages were calculated by SAMtools [98]. The Illumina Genome Analyzer II data of *H. avenae* transcriptomes by Kumar et al. were downloaded from NCBI SRA (ERR414136 for hatched J2 stage and ERR414137 for female stage).

### Quantitative analysis of neuropeptides expression

Total RNA of different life stages of *H. avenae* was extracted using the RNeasy Plus Micro Kit (Qiagen, Germany), which included genomic DNA eliminator columns to remove DNA. The subsequent quantitation of mRNA for FMRFamide-like peptides (FLPs) was performed by quantitative real-time PCR (qPCR) as previously described [78]. Briefly, a SYBR Green based qPCR assay was used to quantify the expression of candidate FLPs after cDNA preparation by QuantiTect Whole Transcriptome Kit (Qiagen, Germany). qPCR was performed using a SYBR Premix Ex Taq (TaKaRa, Japan) in an ABI Prism 7000 instrument (Applied Biosystems, USA). Triplicate PCR reactions for each cDNA sample were completed, and the assay consisted of three technical replicates. Data were analyzed using the 2^−ΔΔCt^ method with *Gapdh* as the endogenous control. The sequences for qPCR primers are as following: *CCN*-*Flp*-*1*, forward 5′-ACGGGCACCTTACGCCAATG-3, reverse 5′-GTTCGGCTCAGAAGCGGACA-3′; *CCN*-*Flp*-*6*, forward 5′-TCTTTGCCGTCTGTTCC-3, reverse 5′-ATTTCGTCGCATCGTC-3′; *CCN*-*Flp*-*12*, forward 5′-TTTCCCTTCCGTCGTTCTC-3, reverse 5′-ACAGCAGTGCCAAAGTCATC-3′; *CCN*-*Flp*-*14*, forward 5′-TGCTTCATCTGCCTCCC-3, reverse 5′-CAATGCCTTCGTTCACCA-3′; *CCN*-*Flp*-*16*, forward 5′-ACCTTCTCCTGCGTTTCG-3, reverse 5′-AAGTTGGGTGACGGTTTGA-3′; *CCN*-*Flp*-*18*, forward 5′-TCCAAAGAATCGCCACA-3, reverse 5′-TACGCCGTTGAGAAAGT-3′; *CCN*-*Gapdh*, forward 5′-AGCGGCACAGAACATCATCC-3, reverse 5′-GGTCCTCCGTGTAGCCCAAA-3′.

## Additional files

**Additional file 1: Figure S1.** The distribution of categories of GO (A) and KEGG pathways (B) annotations for *H. avenae* ESTs (GO terms: Level <3, Percent of genes >5%).

**Additional file 2: Figure S2.** Hierarchical layout of significantly enriched GOs of orthologous identified in *H. avenae* and 7 other nematodes. **a** Enriched GOs of orthologous present in *H. avenae* and all other 7 nematodes (*Globodera pallida, Meloidogyne incognita*, *Meloidogyne hapla*, *Bursaphelenchus xylophilus*, *Caenorhabditis elegans*, *Pristionchus pacifics* and *Ascaris suum*). **b** Enriched GOs of orthologous shared only by *H. avenae* and other plant-parasitic nematodes (*G. pallida, M. incognita*, *M. hapla* and *B. xylophilus*). **c** Enriched GOs of orthologous shared only by *H. avenae* and other sedentary plant-nematodes (*G. pallida, M. incognita* and *M. hapla*). **d** Enriched GOs of orthologous present only in cyst nematodes *H. avenae* and *G. pallida*. Light color represents significant enrichment (*p* < 0.1) and darker color represents more significant enrichment (*p* < 0.05).

**Additional file 3: Table S1.** Comparative analyses of orthologous identified in biological pathways. Orthologous in RNAi pathway **a**, immune responses **b**, dauer/diapause pathway **c** and the synthesis, transportation and metabolism of neurotransmitters **d** were compared among *C. elegans*, *H. avenae* and other 6 nematodes with published genomic data. Cel, *Caenorhabditis elegans*; CCN, Cereal cyst nematode *H. avenae*; Gpa, *Globodera pallida*; Mha, *Meloidogyne hapla*; Min, *Meloidogyne incognita*; Bxy, *Bursaphelenchus xylophilus*; Asu, *Ascaris suum*; Ppa, *Pristionchus pacifics.*

**Additional file 4: Table S2.** Datasets of the nematoda ESTs used in this study. The datasets from NCBI and Nembase 4 databases consist of 1,331,401 ESTs sequences from 45 genuses and 75 species clustered into three life styles. * Life style classification: FLN, free living nematode; PPN, plant parasitic nematode; APN, animal parasitic nematode.

**Additional file 5: Figure S3.** Transcriptomes comparison of *H. avenae* at pre-parasitic and post-parasitic stages. Dot plots show the distributions of contig lengths and abundances in transcriptomes of *H. avenae* before (**a**) and after (**b**) parasitism. **c** MA-plot shows differential expression of genes before and after parasitism. The M is the reads number ratio and the A is the average. Red dots, genes with significantly higher expression at pre-parasitic stages; Blue dots, genes with significantly higher expression at post-parasitic stages (FDR < 0.001). The dotted lines indicate the M values as -1, 0 and 1.

**Additional file 6: Figure S4.** Heat map depicting the expression profiles of crosstalks genes relevant to the KEGG pathways significantly enriched before and after parasitism in *H. avenae*. The first box of each row marks the enrichment of crosstalk at either pre-parasitic stages (purple) or post-parasitic stages (green). Each red box denotes a single gene with higher expression at pre-parasitic stages; each blue box denotes a single gene with higher expression at post-parasitic stages. The darkness of the color is positively correlated with the absolute value of fold change, which is calculated as normalized log_2_(pre-parasitic RPKM/post-parasitic RPKM) with DEGseq.

**Additional file 7: Table S3.** The list of differentially expressed genes in each node from the *H. avenae* KEGG pathways enriched crosstalks. The ‘Log_2_(fold change) Pre/Post’ means the normalized Log_2_(pre-parasitic RPKM/post-parasitic RPKM) of each contig. Both Log_2_(fold change) and Q value threshold were calculated by DEGseq. Some contigs might be presented in multiple crosstalk nodes.

**Additional file 8: Table S4.** Putative phytonematode effectors identified in *H. avenae.* **a** Putative key genes based on blastx searching against other phytonematode effectors. **b** Carbohydrate-active enzymes with homology to those of other phytonematodes. * ^#^ CCN ESTs, number of ESTs in Cereal cyst nematode *H. avenae*. ‘Pre’ means significantly high expression in pre-parasitic stage; ‘Pre*’ means coding by singleton in pre-parasitic library. The singleton might be the gene with low expression and was sequenced only once. CBM, carbohydrate binding module; GH, glycoside hydrolase; GT, glycosyl transferase; PL, polysaccharide lysate. NR means non-redundant GenBank proteins database.

## Abbreviations

ANTs: abundant novel transcripts
BT-PCD: BAX triggered plant programmed cell death
CAZymes: carbohydrate-active enzymes
CCN: cereal cyst nematode
cDNA: complementary DNA
dsRNA: double stranded RNA
EST: expressed sequence tag
FDR: false discovery rate
FLPs: FMRFamide-like peptides
GO: Gene Ontology
KEGG: Kyoto Encyclopedia of Genes and Genomes
KO: KEGG orthologs
NR: non-redundant GenBank proteins database
ORF: open reading frame
RNAi: RNA interference
RPKM: reads per kilo bases per million reads
UTR: untranslated region
