# Supplementary material for: Comparative analysis of pre- and post-parasitic transcriptomes and mining pioneer effectors of Heterodera avenae
Source: Cell Biosci. 2017 Feb 14;7:11. doi: 10.1186/s13578-017-0138-6 (PMC5309974; doi:10.1186/s13578-017-0138-6)

Additional file 1: Figure S1. The distribution of categories of GO (A) and KEGG pathways (B) annotations for *H. avenae* ESTs (GO terms: Level < 3, Percent of genes > 5%).

A

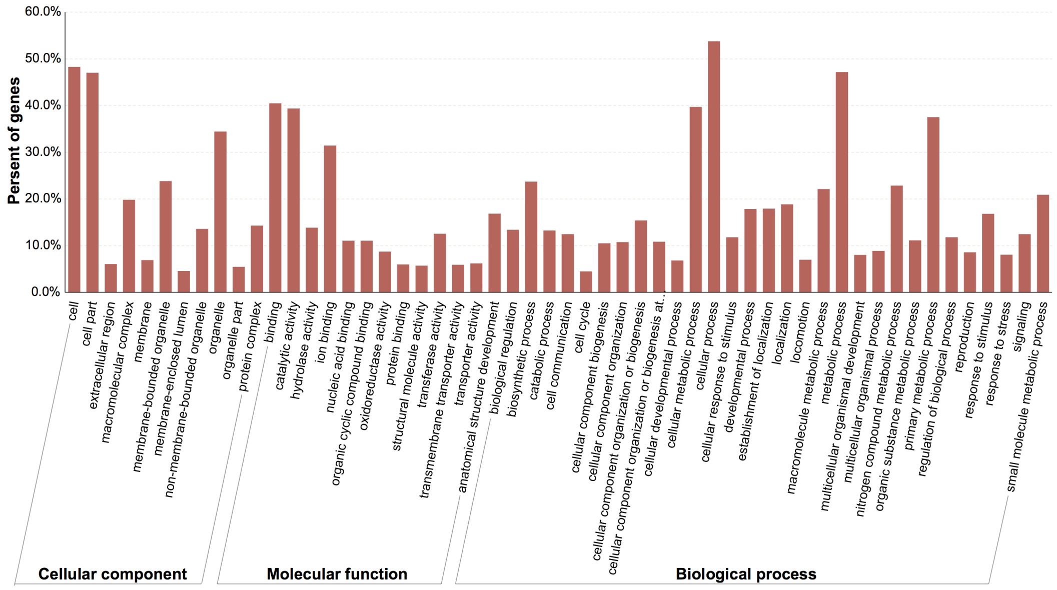

B

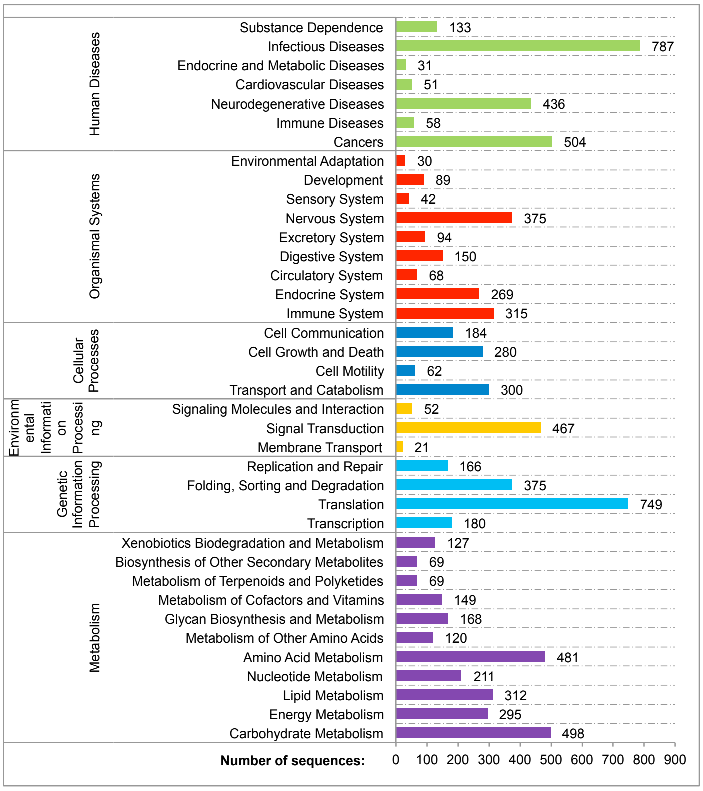

Supplement: Supplementary file 1 — Additional file 1: Figure S1. The distribution of categories of GO (A) and KEGG pathways (B) annotations for H. avenae ESTs (GO terms: Level <3, Percent of genes >5%). [file 13578_2017_138_MOESM1_ESM.pdf]
