# Supplementary material for: Comparative analysis of pre- and post-parasitic transcriptomes and mining pioneer effectors of Heterodera avenae
Source: Cell Biosci. 2017 Feb 14;7:11. doi: 10.1186/s13578-017-0138-6 (PMC5309974; doi:10.1186/s13578-017-0138-6)

Additional file 2: Figure S2. Hierarchical layout of significantly enriched GOs of orthologous identified in *H. avenae* and 7 other nematodes. (**A**) Enriched GOs of orthologous present in *H. avenae* and all other 7 nematodes (*Globodera pallida, Meloidogyne incognita, Meloidogyne hapla, Bursaphelenchus xylophilus, Caenorhabditis elegans, Pristionchus pacifics* and *Ascaris suum*). (**B**) Enriched GOs of orthologous shared only by *H. avenae* and other plant-parasitic nematodes (*G. pallida, M. incognita, M. hapla* and *B. xylophilus*). (**C**) Enriched GOs of orthologous shared only by *H. avenae* and other sedentary plant-nematodes (*G. pallida, M. incognita* and *M. hapla*). (**D**) Enriched GOs of orthologous present only in cyst nematodes *H. avenae* and *G. pallida*. Light color represents significant enrichment ($p < 0.1$) and darker color represents more significant enrichment ($p < 0.05$).

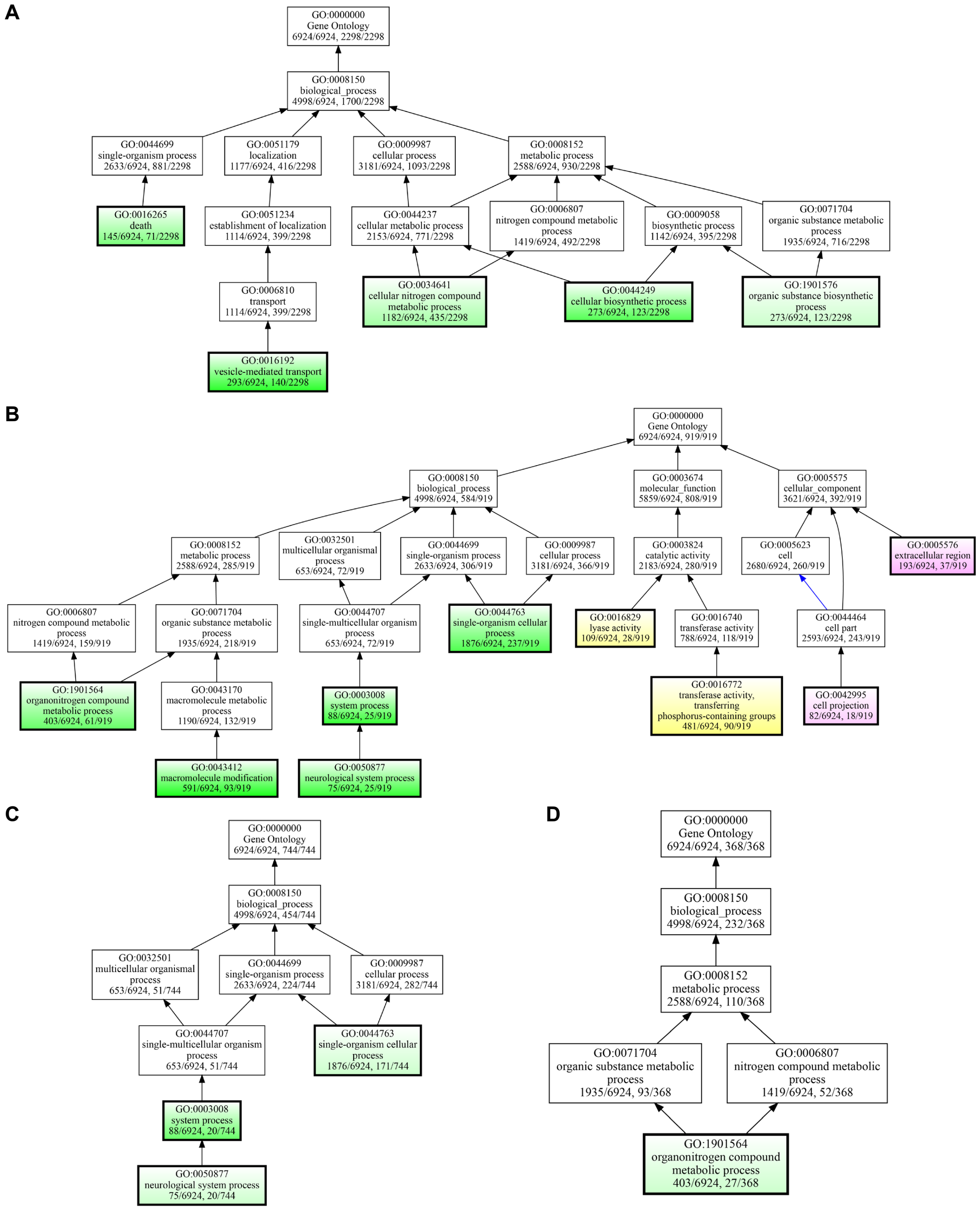

Supplement: Supplementary file 2 — Additional file 2: Figure S2. Hierarchical layout of significantly enriched GOs of orthologous identified in H. avenae and 7 other nematodes. a Enriched GOs of orthologous present in H. avenae and all other 7 nematodes (Globodera pallida, Meloidogyne incognita, Meloidogyne hapla, Bursaphelenchus xylophilus, Caenorhabditis elegans, Pristionchus pacifics and Ascaris suum). b Enriched GOs of orthologous shared only by H. avenae and other plant-parasitic nematodes (G. pallida, M. incognita, M. hapla and B. xylophilus). c Enriched GOs of orthologous shared only by H. avenae and other sedentary plant-nematodes (G. pallida, M. incognita and M. hapla). d Enriched GOs of orthologous present only in cyst nematodes H. avenae and G. pallida. Light color represents significant enrichment (p < 0.1) and darker color represents more significant enrichment (p < 0.05). [file 13578_2017_138_MOESM2_ESM.pdf]
