# Supplementary material for: Comparative analysis of pre- and post-parasitic transcriptomes and mining pioneer effectors of Heterodera avenae
Source: Cell Biosci. 2017 Feb 14;7:11. doi: 10.1186/s13578-017-0138-6 (PMC5309974; doi:10.1186/s13578-017-0138-6)
Supplement: Supplementary file 3 — Additional file 3: Table S1. Comparative analyses of orthologous identified in biological pathways. Orthologous in RNAi pathway a, immune responses b, dauer/diapause pathway c and the synthesis, transportation and metabolism of neurotransmitters d were compared among C. elegans, H. avenae and other 6 nematodes with published genomic data. Cel, Caenorhabditis elegans; CCN, Cereal cyst nematode H. avenae; Gpa, Globodera pallida; Mha, Meloidogyne hapla; Min, Meloidogyne incognita; Bxy, Bursaphelenchus xylophilus; Asu, Ascaris suum; Ppa, Pristionchus pacifics. [file 13578_2017_138_MOESM3_ESM.pdf]

Additional file 3: Table S1. Comparative analyses of orthologous identified in biological pathways. Orthologous in RNAi pathway (A), immune responses (B), dauer/diapause pathway (C) and the synthesis, transportation and metabolism of neurotransmitters (D) were compared among *C. elegans*, *H. avenae* and other 6 nematodes with published genomic data. Cel, *Caenorhabditis elegans*; CCN, Cereal cyst nematode *H. avenae*; Gpa, *Globodera pallida*; Mha, *Meloidogyne hapla*; Min, *Meloidogyne incognita*; Bxy, *Bursaphelenchus xylophilus*; Asu, *Ascaris suum*; Ppa, *Pristionchus pacifics*.

Table S1A. Comparison of orthologous identified in RNAi pathway among *C. elegans*, *H. avenae* and other 6 nematodes with published genomic data.

Gene function	Cel	CCN	Gpa	Mha	Min	Bxy	Asu	Ppa
	drh-3	+	+	+	+	+	+	+
	drsh-1	+	+	+	+	+	+	
	xpo-1	+	+	+	+	+	+	+
	xpo-2	+	+	+	+	+	+	+
**Small RNA biosynthetic proteins**	*dcr-1*	+	+	+	+	+	+	+
	drh-1			+	+	+	+	
	pash-1	+	+	+	+	+	+	+
	rde-4							
	xpo-3	+					+	
	smg-2	+	+	+	+	+	+	+
	smg-6	+	+	+	+	+	+	+
	ego-1	+	+	+	+	+	+	+
**Amplification**	*rrf-3*					+	+	+
	rrf-1							
	smg-5							
	rsd-2							
	rsd-3	+	+	+	+	+	+	+
**Spreading**	*sid-1*						+	
	rsd-6							
	sid-2							
	alg-1	+	+	+	+	+	+	+
	wago-1	+	+	+	+	+	+	+
	C04F12.1	+	+	+	+		+	+
	wago-4	+	+	+	+	+	+	+
	alg-4					+	+	+
	rde-1	+	+	+	+	+	+	+
	hrde-1	+	+	+	+	+	+	+
	ppw-1							
	csr-1	+	+	+	+	+		
	ppw-2	+	+	+	+	+	+	+
	sago-1							
**Argonautes**	*alg-3*					+	+	+
	wago-10	+	+	+	+	+	+	+
	alg-2							
	ergo-1							
	prg-1							+
	wago-2	+	+	+	+	+	+	+
	hpo-24							
	nrde-3	+	+	+	+	+	+	+
	sago-2							
	T23B3.2						+	+
	wago-11	+	+	+	+	+	+	+
	wago-5	+	+	+	+	+	+	+
	prg-2							+
	tsn-1	+	+	+	+	+	+	+
**Other RISC components**	*ain-1*	+		+	+	+	+	
	vig-1					+	+	+
	ain-2	+	+					

**RNAi inhibitors**	*eri-1*	+	+	+	+		+	+
	xrn-2	+	+	+	+	+	+	+
	adr-2						+	+
	xrn-1						+	+
	adr-1					+	+	
	lin-15b							
	eri-5							
	eri-6							
	eri-7	+				+	+	
	eri-3							
**Nuclear RNAi effectors**	*mut-7*	+	+	+		+	+	+
	cid-1	+	+	+	+	+	+	
	ekl-1	+	+	+	+	+	+	+
	gfl-1	+	+		+	+	+	
	mes-2	+	+	+	+	+	+	
	ekl-4	+		+	+	+	+	+
	mes-6	+	+	+		+	+	
	rha-1	+	+	+	+	+	+	
	ekl-6					+	+	+
	zfp-1						+	+
	mut-2							+
	ekl-5							
	mes-3							
	mut-16							
	rde-2							

Cel, *Caenorhabditis elegans*; CCN, Cereal cyst nematode *H. avenae*; Gpa, *Globodera pallida*; Mha, *Meloidogyne hapla*; Min, *Meloidogyne incognita*; Bxy, *Bursaphelenchus xylophilus*; Asu, *Ascaris suum*; Ppa, *Pristionchus pacifics*.

Table S1B. Comparison of orthologous identified in immune responses among *C. elegans*, *H. avenae* and other 6 nematodes with published genomic data.

Immune signaling	Cel	CCN	Gpa	Mha	Min	Bxy	Asu	Ppa
**DBL-1/TGF-beta signalling pathway**	*dbl-1*	+	+	+	+	+	+	+
	sma-2	+	+	+	+	+	+	+
	sma-3	+	+	+	+	+	+	
	sma-4	+	+	+	+	+	+	+
**ERK MAPK signalling pathway**	*lin-45*	+	+	+	+	+	+	+
	mak-2	+	+	+	+	+	+	+
	mpk-1	+	+	+	+	+	+	
**P38 MAPK signalling pathway**	*nsy-1*	+	+	+	+	+	+	+
	pmk-1	+	+	+		+	+	+
	sek-1	+	+	+	+	+	+	
	tir-1	+	+	+	+	+	+	+
**Toll signalling pathway**	*tol-1*	+	+	+	+	+	+	
	trf-1	+				+	+	+
	ikb-1						+	+
	plk-1	+	+	+	+	+	+	
**Insulin (DAF-2/DAF-16) signaling pathway**	*age-1*	+		+	+	+	+	+
	pdk-1	+	+	+	+	+	+	+
	akt-1	+	+	+	+	+	+	+
	akt-2	+	+	+	+	+	+	+
	daf-16	+	+	+		+	+	
	daf-2	+	+	+	+	+	+	+

Cel, *Caenorhabditis elegans*; CCN, Cereal cyst nematode *H. avenae*; Gpa, *Globodera pallida*; Mha, *Meloidogyne hapla*; Min, *Meloidogyne incognita*; Bxy, *Bursaphelenchus xylophilus*; Asu, *Ascaris suum*; Ppa, *Pristionchus pacifics*.

Table S1C. Comparison of orthologous identified in dauer/diapause pathway among *C. elegans*, *H. avenae* and *G. pallida*.

Dauer pathway	Protein	Cel	CCN	Gpa
**Guanylyl cyclase pathway**	Transmembrane guanylate cyclase	*daf-11*	+	+
	cGMP-gated channel	*tax-2*	+	+
	cGMP-gated channel	*tax-4*	+	+
**TGFβ-like**	TGF-β type I receptor	*daf-1*	+	+
	SMAD transcription factor	*daf-3*	+	+
	TGFβ type II receptor	*daf-4*	+	+
	Proline rich protein	*daf-5*		
	BMP/TGF-β	*daf-7*	+	+
	SMAD transcription factor	*daf-8*		
	SMAD transcription factor	*daf-14*		
	Glutamine rich protein	*scd-1*	+	+
	Tyrosine kinase	*scd-2*	+	+
	Zn-finger protein	*bra-1*	+	
	Tyrosine kinase	*cam-1*	+	+
	cGMP-dependent protein kinase	*egl-4*	+	+
**Insulin/IGF**	Insulin receptor	*daf-2*	+	+
	Ortholog RAPTOR protein	*daf-15*	+	+
	FOXO transcription factor	*daf-16*	+	+
	Phosphoinositide 3-phosphatase PTEN	*daf-18*	+	
	β-insulin	*daf-28*		
	Phosphoinositide 3-kinase	*age-1*	+	
	3-phophoinositide-dependent kinase	*pdk-1*	+	+
	Serine/threonine kinase	*akt-1*	+	+
	Serine/threonine kinase	*akt-2*	+	+
	Serine/threonine kinase	*sgk-1*	+	
**Steroid hormone pathway**	Cytochrome P450	*daf-9*	+	+
	Nuclear receptor	*daf-12*	+	+
	Rieske oxygenase, hormone pathway	*daf-36*	+	
**Other processes**	amphid morphology	*daf-6*		+
	WD-WAA rep	*daf-10*	+	+
	RFX transcription factor	*daf-19*	+	+
	HSP-90	*daf-21*		+

Cel, *Caenorhabditis elegans*; CCN, Cereal cyst nematode *H. avenae*; Gpa, *Globodera pallida*.

Table S1D. Comparison of orthologous identified in the synthesis, transportation and metabolism of neurotransmitters among *C. elegans*, *H. avenae* and other 4 plant-parasitic nematodes.

Neurotransmitter	Gene function	Cel	CCN	Gpa	Mha	Min	Bxy
**Acetylcholine**	Choline acetyltransferase	*cha-1*	+	+	+	+	+
	Synaptic acetylcholine transporter	*unc-17*	+	+	+		+
	Choline transporter	*cho-1*	+	+	+		+
	Post-synaptic transporter	*snf-6*	+	+	+	+	+
	Acetylcholinesterase	*ace-1*	+	+	+		+
	Acetylcholinesterase	*ace-2*	+	+		+	+
	Acetylcholinesterase	*ace-3*	+	+	+	+	+
	Acetylcholinesterase	*ace-4*					
**Serotonin**	Tryptophan hydroxylase	*tph-1*		+	+		+
	GTP-cyclohydrolase I	*cat-4*	+	+	+		+
	Aromatic AA decarboxylase	*bas-1*	+	+	+		+
	Vesicular monoamine transporter	*cat-1*	+	+	+		+
	Serotonin reuptake transporter	*mod-5*	+	+	+	+	+
	Monoamine oxidase	*amx-1*					+
	Monoamine oxidase	*amx-2*					
	Monoamine oxidase	*amx-3*					
**Dopamine**	Tyrosine hydroxylase	*cat-2*					+
	Dopamine reuptake transporter	*dat-1*	+	+	+		+
**Tyramine**	Tyrosine decarboxylase	*tdc-1*	+	+	+	+	+
**Octopamine**	Tyramine β-hydroxylase	*tbh-1*	+	+	+	+	+
**Glutamate**	Vesicular glutamate transporter	*eat-4*	+	+	+	+	+
	Plasma membrane glutamate transporter	*glt-1*	+	+	+	+	+
**GABA**	Glutamate decarboxylase	*unc-25*	+	+	+	+	+
	Esicular GABA transporter	*unc-47*	+	+	+	+	+
	GABA transporter	*snf-11*	+	+	+	+	+
	GABA transaminase	*gta-1*	+	+	+	+	+

Cel, *Caenorhabditis elegans*; CCN, Cereal cyst nematode *H. avenae*; Gpa, *Globodera pallida*; Mha, *Meloidogyne hapla*; Min, *Meloidogyne incognita*; Bxy, *Bursaphelenchus xylophilus*.
