# Supplementary material for: Comparative analysis of pre- and post-parasitic transcriptomes and mining pioneer effectors of Heterodera avenae
Source: Cell Biosci. 2017 Feb 14;7:11. doi: 10.1186/s13578-017-0138-6 (PMC5309974; doi:10.1186/s13578-017-0138-6)
Supplement: Supplementary file 4 — Additional file 4: Table S2. Datasets of the nematoda ESTs used in this study. The datasets from NCBI and Nembase 4 databases consist of 1,331,401 ESTs sequences from 45 genuses and 75 species clustered into three life styles. * Life style classification: FLN, free living nematode; PPN, plant parasitic nematode; APN, animal parasitic nematode. [file 13578_2017_138_MOESM4_ESM.pdf]

Additional file 4: Table S2. Datasets of the nematoda ESTs used in this study. The datasets from NCBI and Nembase 4 databases consist of 1,331,401 ESTs sequences from 45 genuses and 75 species clustered into three life styles. * Life style classification: FLN, free living nematode; PPN, plant parasitic nematode; APN, animal parasitic nematode.

Life style classification*	Genus	Species	Texid	Num. of ESTs
FLN	*Caenorhabditis*	*Caenorhabditis briggsae*	6238	2424
FLN	*Caenorhabditis*	*Caenorhabditis elegans*	6239	396687
FLN	*Caenorhabditis*	*Caenorhabditis remanei*	31234	20292
FLN	*Zeldia*	*Zeldia punctata*	49351	391
FLN	*Pristionchus*	*Pristionchus pacificus*	54126	37470
FLN	*Caenorhabditis*	*Caenorhabditis brenneri*	135651	30074
FLN	*Panagrolaimus*	*Panagrolaimus davidi*	227884	95141
FLN	*Caenorhabditis*	*Caenorhabditis japonica*	281687	33050
FLN	*Panagrolaimus*	*Panagrolaimus superbus*	310955	7609
FLN	*Caenorhabditis*	*Caenorhabditis sp. 5 AC-2008*	497871	3868
FLN	*Plectus*	*Plectus murrayi*	538668	2591
PPN	*Meloidogyne*	*Meloidogyne javanica*	6303	7587
PPN	*Meloidogyne*	*Meloidogyne arenaria*	6304	5042
PPN	*Meloidogyne*	*Meloidogyne hapla*	6305	24452
PPN	*Meloidogyne*	*Meloidogyne incognita*	6306	63838
PPN	*Bursaphelenchus*	*Bursaphelenchus mucronatus*	6325	3193
PPN	*Bursaphelenchus*	*Bursaphelenchus xylophilus*	6326	14059
PPN	*Globodera*	*Globodera rostochiensis*	31243	11851
PPN	*Heterodera*	*Heterodera avenae*	34510	27766
PPN	*Globodera*	*Globodera pallida*	36090	9020
PPN	*Pratylenchus*	*Pratylenchus penetrans*	45929	1916
PPN	*Pratylenchus*	*Pratylenchus vulnus*	45931	5812
PPN	*Pratylenchus*	*Pratylenchus thornei*	45935	6667
PPN	*Xiphinema*	*Xiphinema index*	46003	9351
PPN	*Radopholus*	*Radopholus similis*	46012	7382
PPN	*Heterodera*	*Heterodera glycines*	51029	24444
PPN	*Meloidogyne*	*Meloidogyne chitwoodi*	59747	12218
PPN	*Aphelenchus*	*Aphelenchus avenae*	70226	5119
PPN	*Heterodera*	*Heterodera schachtii*	97005	2812
PPN	*Anguina*	*Anguina tritici*	166006	59
PPN	*Globodera*	*Globodera mexicana*	182293	17
PPN	*Meloidogyne*	*Meloidogyne paranaensis*	189293	3710
PPN	*Rotylenchulus*	*Rotylenchulus reniformis*	239373	2004
PPN	*Ditylenchus*	*Ditylenchus africanus*	490491	4847
PPN	*Dictyocaulus*	*Ditylenchus destructor*	166010	9800
APN	*Strongyloides*	*Strongyloides stercoralis*	6248	11392
APN	*Ascaris*	*Ascaris lumbricoides*	6252	1822
APN	*Ascaris*	*Ascaris suum*	6253	56616
APN	*Toxocara*	*Toxocara canis*	6265	5089
APN	*Anisakis*	*Anisakis simplex*	6269	475
APN	*Brugia*	*Brugia malayi*	6279	26215
APN	*Brugia*	*Brugia pahangi*	6280	28
APN	*Onchocerca*	*Onchocerca volvulus*	6282	14974
APN	*Dirofilaria*	*Dirofilaria immitis*	6287	4005
APN	*Haemonchus*	*Haemonchus contortus*	6289	21975
APN	*Wuchereria*	*Wuchereria bancrofti*	6293	4847
APN	*Angiostrongylus*	*Angiostrongylus cantonensis*	6313	2622
APN	*Ostertagia*	*Ostertagia ostertagi*	6317	7006
APN	*Trichinella*	*Trichinella spiralis*	6334	25301
APN	*Trichinella*	*Trichinella pseudospiralis*	6337	17330
APN	*Loa*	*Loa loa*	7209	4173
APN	*Nippostrongylus*	*Nippostrongylus brasiliensis*	27835	14686
APN	*Ancylostoma*	*Ancylostoma caninum*	29170	80905
APN	*Dictyocaulus*	*Dictyocaulus viviparus*	29172	7917
APN	*Strongyloides*	*Strongyloides ratti*	34506	27366
APN	*Steinernema*	*Steinernema carpocapsae*	34508	2218
APN	*Heterorhabditis*	*Heterorhabditis bacteriophora*	37862	53614
APN	*Trichostrongylus*	*Trichostrongylus vitrinus*	40352	368
APN	*Litomosoides*	*Litomosoides sigmodontis*	42156	2699
APN	*Onchocerca*	*Onchocerca ochengi*	42157	60
APN	*Teladorsagia*	*Teladorsagia circumcincta*	45464	6061

APN	*Necator*	*Necator americanus*	51031	6694
APN	*Steinernema*	*Steinernema feltiae*	52066	83
APN	*Ancylostoma*	*Ancylostoma ceylanicum*	53326	10651
APN	*Toxascaris*	*Toxascaris leonina*	59264	439
APN	*Oesophagostomum*	*Oesophagostomum dentatum*	61180	299
APN	*Trichuris*	*Trichuris muris*	70415	7102
APN	*Strongyloides*	*Strongyloides venezuelensis*	75913	408
APN	*Parastrongyloides*	*Parastrongyloides trichosuri*	131310	7963
APN	*Parelaphostrongylus*	*Parelaphostrongylus tenuis*	148309	99
APN	*Trichuris*	*Trichuris vulpis*	219738	3063
APN	*Mecistocirrus*	*Mecistocirrus digitatus*	237660	1
APN	*Ancylostoma*	*Ancylostoma braziliense*	369059	20
APN	*Onchocerca*	*Onchocerca flexuosa*	387005	2124
APN	Anguillicoloides	*Anguillicoloides crassus*	620897	128
