# Supplementary material for: Comparative analysis of pre- and post-parasitic transcriptomes and mining pioneer effectors of Heterodera avenae
Source: Cell Biosci. 2017 Feb 14;7:11. doi: 10.1186/s13578-017-0138-6 (PMC5309974; doi:10.1186/s13578-017-0138-6)

**Additional file 5: Figure S3.** Transcriptomes comparison of *H. avenae* at pre-parasitic and post-parasitic stages. (**A-B**) Dot plots show the distributions of contig lengths and abundances in transcriptomes of *H. avenae* before (**A**) and after (**B**) parasitism. (**C**) MA-plot shows differential expression of genes before and after parasitism. The M is the reads number ratio and the A is the average. Red dots, genes with significantly higher expression at pre-parasitic stages; Blue dots, genes with significantly higher expression at post-parasitic stages (FDR < 0.001). The dotted lines indicate the M values as -1, 0 and 1.

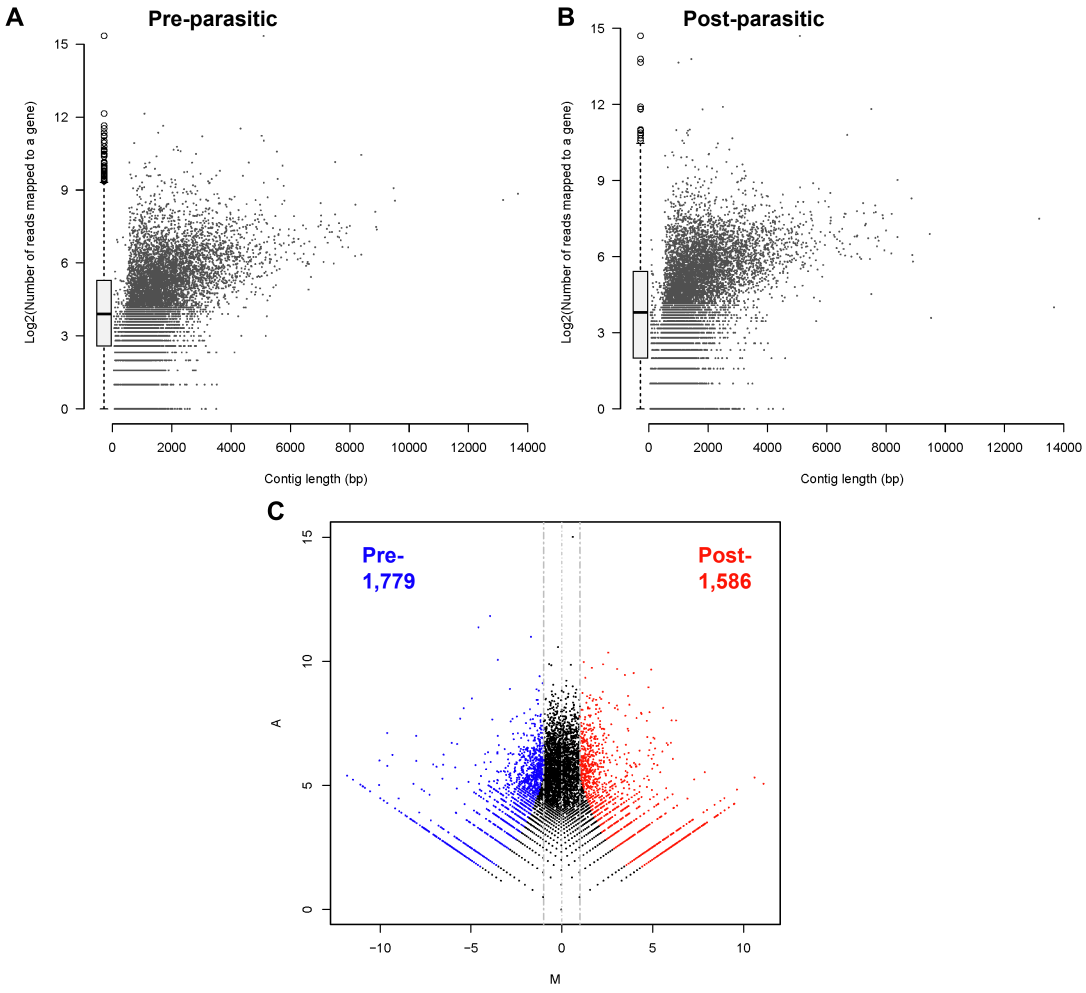

Supplement: Supplementary file 5 — Additional file 5: Figure S3. Transcriptomes comparison of H. avenae at pre-parasitic and post-parasitic stages. Dot plots show the distributions of contig lengths and abundances in transcriptomes of H. avenae before (a) and after (b) parasitism. c MA-plot shows differential expression of genes before and after parasitism. The M is the reads number ratio and the A is the average. Red dots, genes with significantly higher expression at pre-parasitic stages; Blue dots, genes with significantly higher expression at post-parasitic stages (FDR < 0.001). The dotted lines indicate the M values as -1, 0 and 1. [file 13578_2017_138_MOESM5_ESM.pdf]
