# Supplementary material for: Comparative analysis of pre- and post-parasitic transcriptomes and mining pioneer effectors of Heterodera avenae
Source: Cell Biosci. 2017 Feb 14;7:11. doi: 10.1186/s13578-017-0138-6 (PMC5309974; doi:10.1186/s13578-017-0138-6)

Additional file 6: Figure S4. Heat map depicting the expression profiles of crosstalks genes relevant to the KEGG pathways significantly enriched before and after parasitism in *H. avenae*. The first box of each row marks the enrichment of crosstalk at either pre-parasitic stages (purple) or post-parasitic stages (green). Each red box denotes a single gene with higher expression at pre-parasitic stages; each blue box denotes a single gene with higher expression at post-parasitic stages. The darkness of the color is positively correlated with the absolute value of fold change, which is calculated as normalized $\log_2$(pre-parasitic RPKM/post-parasitic RPKM) with DEGseq.

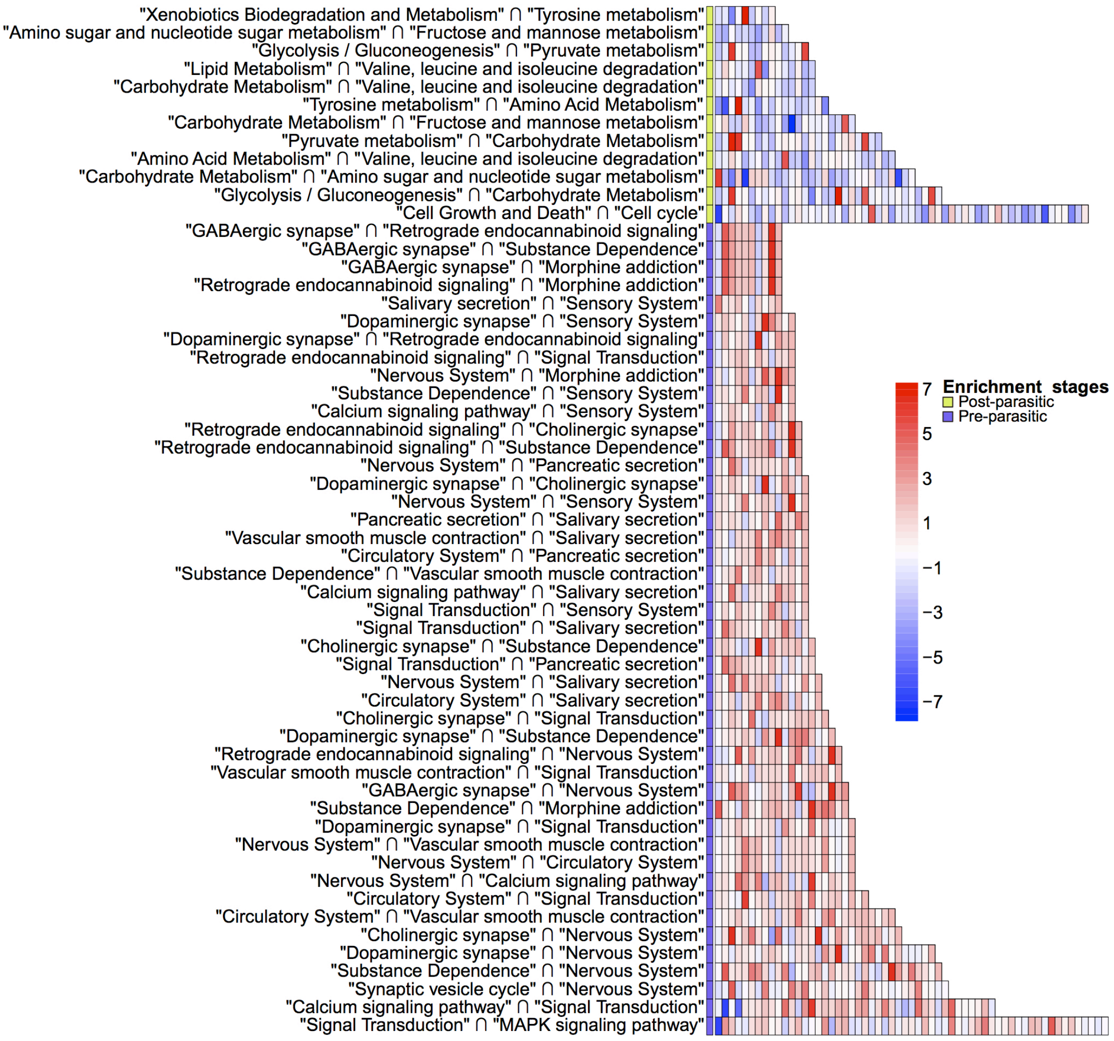

Supplement: Supplementary file 6 — Additional file 6: Figure S4. Heat map depicting the expression profiles of crosstalks genes relevant to the KEGG pathways significantly enriched before and after parasitism in H. avenae. The first box of each row marks the enrichment of crosstalk at either pre-parasitic stages (purple) or post-parasitic stages (green). Each red box denotes a single gene with higher expression at pre-parasitic stages; each blue box denotes a single gene with higher expression at post-parasitic stages. The darkness of the color is positively correlated with the absolute value of fold change, which is calculated as normalized log2(pre-parasitic RPKM/post-parasitic RPKM) with DEGseq. [file 13578_2017_138_MOESM6_ESM.pdf]
