# Supplementary material for: Comparative analysis of pre- and post-parasitic transcriptomes and mining pioneer effectors of Heterodera avenae
Source: Cell Biosci. 2017 Feb 14;7:11. doi: 10.1186/s13578-017-0138-6 (PMC5309974; doi:10.1186/s13578-017-0138-6)
Supplement: Supplementary file 7 — Additional file 7: Table S3. The list of differentially expressed genes in each node from the H. avenae KEGG pathways enriched crosstalks. The ‘Log2(fold change) Pre/Post’ means the normalized Log2(pre-parasitic RPKM/post-parasitic RPKM) of each contig. Both Log2(fold change) and Q value threshold were calculated by DEGseq. Some contigs might be presented in multiple crosstalk nodes. [file 13578_2017_138_MOESM7_ESM.pdf]

Additional file 7: Table S3. The list of differentially expressed genes in each node from the *H. avenae* KEGG pathways enriched crosstalks. The 'Log$_2$(Fold change) Pre/Post' means the normalized Log$_2$(pre-parasitic RPKM/post-parasitic RPKM) of each contig. Both Log$_2$(Fold change) and Q value threshold were calculated by DEGseq. Some contigs might be presented in multiple crosstalk nodes.

Crosstalks type	Crosstalks node	KO number of KEGG	CCN contig	Log$_2$(Fold change) Pre/Post	Q value
Pre	Calcium signaling pathway	K02183	isotig10702	3.67	5.23E-04
Pre	Calcium signaling pathway	K02677	isotig08175	1.89	4.34E-15
Pre	Calcium signaling pathway	K04131	isotig15521	4.29	1.12E-05
Pre	Calcium signaling pathway	K04163	isotig18466	4.43	6.94E-04
Pre	Calcium signaling pathway	K04345	isotig08250	-1.94	3.80E-12
Pre	Calcium signaling pathway	K04345	isotig02324	1.78	2.56E-20
Pre	Calcium signaling pathway	K04348	isotig05039	3.1	1.80E-14
Pre	Calcium signaling pathway	K04361	isotig14617	-6.42	5.25E-11
Pre	Calcium signaling pathway	K04962	isotig13331	2.69	9.99E-13
Pre	Calcium signaling pathway	K05327	isotig13387	1.71	5.66E-04
Pre	Calcium signaling pathway	K05858	isotig03047	1.1	2.44E-07
Pre	Calcium signaling pathway	K05860	isotig13702	2.78	1.24E-04
Pre	Calcium signaling pathway	K05863	isotig16228	-5.35	1.31E-10
Pre	Calcium signaling pathway	K05863	isotig16053	2.25	1.56E-116
Pre	Calcium signaling pathway	K05863	isotig16273	4.71	1.20E-48
Pre	Calcium signaling pathway	K08049	isotig02947	2	3.38E-21
Pre	Calcium signaling pathway	K13755	isotig08310	4.29	1.12E-05
Pre	Calcium signaling pathway	K16056	isotig10128	-1.61	1.66E-04
Pre	Cholinergic synapse	K02677	isotig08175	1.89	4.34E-15
Pre	Cholinergic synapse	K04131	isotig15521	4.29	1.12E-05
Pre	Cholinergic synapse	K04345	isotig08250	-1.94	3.80E-12
Pre	Cholinergic synapse	K04345	isotig02324	1.78	2.56E-20
Pre	Cholinergic synapse	K04368	isotig05365	1.42	9.34E-05
Pre	Cholinergic synapse	K04539	isotig08600	2.97	2.65E-05
Pre	Cholinergic synapse	K04630	isotig08244	-1.29	4.52E-07
Pre	Cholinergic synapse	K04926	isotig04414	2.12	7.18E-05

Pre	Cholinergic synapse	K05858	isotig03047	1.1	2.44E-07
Pre	Cholinergic synapse	K08049	isotig02947	2	3.38E-21
Pre	Cholinergic synapse	K14387	isotig16478	5.36	3.24E-06
Pre	Cholinergic synapse	K14636	isotig14851	4.06	5.82E-05
Pre	Circulatory System	K00412	isotig12250	4.56	4.01E-04
Pre	Circulatory System	K01047	isotig04965	1.17	4.69E-04
Pre	Circulatory System	K01539	isotig13411	-1.51	1.88E-04
Pre	Circulatory System	K02183	isotig10702	3.67	5.23E-04
Pre	Circulatory System	K02263	isotig19203	-3.73	4.35E-04
Pre	Circulatory System	K02265	isotig19199	-2.28	4.50E-06
Pre	Circulatory System	K02677	isotig08175	1.89	4.34E-15
Pre	Circulatory System	K04345	isotig08250	-1.94	3.80E-12
Pre	Circulatory System	K04345	isotig02324	1.78	2.56E-20
Pre	Circulatory System	K04368	isotig05365	1.42	9.34E-05
Pre	Circulatory System	K04936	isotig13985	3.93	1.32E-07
Pre	Circulatory System	K04962	isotig13331	2.69	9.99E-13
Pre	Circulatory System	K05858	isotig03047	1.1	2.44E-07
Pre	Circulatory System	K06068	isotig04374	1.73	1.66E-11
Pre	Circulatory System	K06068	isotig03922	1.25	5.41E-07
Pre	Circulatory System	K08049	isotig02947	2	3.38E-21
Pre	Circulatory System	K09290	isotig12004	4.39	2.33E-237
Pre	Circulatory System	K12319	isotig15721	2.97	1.54E-06
Pre	Circulatory System	K12323	isotig08178	1.53	3.89E-08
Pre	Dopaminergic synapse	K02183	isotig10702	3.67	5.23E-04
Pre	Dopaminergic synapse	K02677	isotig08175	1.89	4.34E-15
Pre	Dopaminergic synapse	K04345	isotig08250	-1.94	3.80E-12
Pre	Dopaminergic synapse	K04345	isotig02324	1.78	2.56E-20
Pre	Dopaminergic synapse	K04348	isotig05039	3.1	1.80E-14
Pre	Dopaminergic synapse	K04440	isotig14539	-1.19	1.17E-05
Pre	Dopaminergic synapse	K04539	isotig08600	2.97	2.65E-05
Pre	Dopaminergic synapse	K04630	isotig08244	-1.29	4.52E-07
Pre	Dopaminergic synapse	K05858	isotig03047	1.1	2.44E-07

Pre	Dopaminergic synapse	K08155	isotig10076	3.97	5.09E-11
Pre	Dopaminergic synapse	K11583	isotig05144	1.21	6.68E-07
Pre	GABAergic synapse	K01425	isotig16061	4.78	1.36E-04
Pre	GABAergic synapse	K01915	isotig11444	-2.66	5.78E-06
Pre	GABAergic synapse	K02677	isotig08175	1.89	4.34E-15
Pre	GABAergic synapse	K04345	isotig02324	1.78	2.56E-20
Pre	GABAergic synapse	K04345	isotig08250	-1.94	3.80E-12
Pre	GABAergic synapse	K04539	isotig08600	2.97	2.65E-05
Pre	GABAergic synapse	K05034	isotig13994	3.29	1.32E-13
Pre	GABAergic synapse	K08049	isotig02947	2	3.38E-21
Pre	GABAergic synapse	K15015	isotig14774	4.56	4.01E-04
Pre	GABAergic synapse	K15375	isotig07308	2.89	1.77E-15
Pre	MAPK signaling pathway	K01047	isotig04965	1.17	4.69E-04
Pre	MAPK signaling pathway	K02677	isotig08175	1.89	4.34E-15
Pre	MAPK signaling pathway	K03099	isotig08975	2.25	6.33E-22
Pre	MAPK signaling pathway	K04345	isotig02324	1.78	2.56E-20
Pre	MAPK signaling pathway	K04345	isotig08250	-1.94	3.80E-12
Pre	MAPK signaling pathway	K04348	isotig05039	3.1	1.80E-14
Pre	MAPK signaling pathway	K04358	isotig11638	3.24	1.87E-06
Pre	MAPK signaling pathway	K04361	isotig14617	-6.42	5.25E-11
Pre	MAPK signaling pathway	K04364	isotig17494	-1.57	6.90E-04
Pre	MAPK signaling pathway	K04368	isotig05365	1.42	9.34E-05
Pre	MAPK signaling pathway	K04375	isotig13308	-1.03	4.75E-04
Pre	MAPK signaling pathway	K04392	isotig15866	-1.32	3.14E-05
Pre	MAPK signaling pathway	K04440	isotig14539	-1.19	1.17E-05
Pre	MAPK signaling pathway	K04459	isotig16388	5.14	1.78E-09
Pre	MAPK signaling pathway	K04459	isotig15433	1.81	7.06E-06
Pre	MAPK signaling pathway	K04463	isotig14730	1.78	6.09E-04
Pre	MAPK signaling pathway	K04674	isotig14825	-1.92	4.30E-05
Pre	MAPK signaling pathway	K05315	isotig14666	3.73	1.21E-06
Pre	MAPK signaling pathway	K05316	isotig05441	4	3.44E-21
Pre	Morphine addiction	K01120	isotig08927	3.08	9.95E-13

Pre	Morphine addiction	K01120	isotig11262	3.56	6.18E-06
Pre	Morphine addiction	K02677	isotig08175	1.89	4.34E-15
Pre	Morphine addiction	K04345	isotig02324	1.78	2.56E-20
Pre	Morphine addiction	K04345	isotig08250	-1.94	3.80E-12
Pre	Morphine addiction	K04539	isotig08600	2.97	2.65E-05
Pre	Morphine addiction	K08049	isotig02947	2	3.38E-21
Pre	Morphine addiction	K13296	isotig04542	2.45	2.79E-25
Pre	Morphine addiction	K13755	isotig08310	4.29	1.12E-05
Pre	Morphine addiction	K15015	isotig14774	4.56	4.01E-04
Pre	Nervous System	K01047	isotig04965	1.17	4.69E-04
Pre	Nervous System	K01115	isotig13270	-1.56	1.95E-09
Pre	Nervous System	K01425	isotig16061	4.78	1.36E-04
Pre	Nervous System	K01528	isotig02826	1.27	4.00E-18
Pre	Nervous System	K01915	isotig11444	-2.66	5.78E-06
Pre	Nervous System	K02152	isotig19022	3.67	2.07E-06
Pre	Nervous System	K02183	isotig10702	3.67	5.23E-04
Pre	Nervous System	K02677	isotig08175	1.89	4.34E-15
Pre	Nervous System	K03099	isotig08975	2.25	6.33E-22
Pre	Nervous System	K04131	isotig15521	4.29	1.12E-05
Pre	Nervous System	K04153	isotig10656	2.47	9.12E-04
Pre	Nervous System	K04163	isotig18466	4.43	6.94E-04
Pre	Nervous System	K04345	isotig02324	1.78	2.56E-20
Pre	Nervous System	K04345	isotig08250	-1.94	3.80E-12
Pre	Nervous System	K04348	isotig05039	3.1	1.80E-14
Pre	Nervous System	K04364	isotig17494	-1.57	6.90E-04
Pre	Nervous System	K04368	isotig05365	1.42	9.34E-05
Pre	Nervous System	K04392	isotig15866	-1.32	3.14E-05
Pre	Nervous System	K04440	isotig14539	-1.19	1.17E-05
Pre	Nervous System	K04463	isotig14730	1.78	6.09E-04
Pre	Nervous System	K04505	isotig03446	1.03	3.58E-08
Pre	Nervous System	K04539	isotig08600	2.97	2.65E-05
Pre	Nervous System	K04630	isotig08244	-1.29	4.52E-07

Pre	Nervous System	K04646	isotig13231	-1.75	5.92E-26
Pre	Nervous System	K04926	isotig04414	2.12	7.18E-05
Pre	Nervous System	K05034	isotig13994	3.29	1.32E-13
Pre	Nervous System	K05858	isotig03047	1.1	2.44E-07
Pre	Nervous System	K06619	isotig13791	1.56	1.66E-04
Pre	Nervous System	K07293	isotig01787	1.71	3.33E-24
Pre	Nervous System	K07413	isotig15601	-4.12	4.65E-05
Pre	Nervous System	K07882	isotig15140	3.85	4.82E-10
Pre	Nervous System	K08049	isotig02947	2	3.38E-21
Pre	Nervous System	K08155	isotig10076	3.97	5.09E-11
Pre	Nervous System	K11583	isotig05144	1.21	6.68E-07
Pre	Nervous System	K12302	isotig17247	4.14	3.35E-05
Pre	Nervous System	K12319	isotig15721	2.97	1.54E-06
Pre	Nervous System	K12460	isotig05593	1.08	2.43E-05
Pre	Nervous System	K14387	isotig16478	5.36	3.24E-06
Pre	Nervous System	K14636	isotig14851	4.06	5.82E-05
Pre	Nervous System	K15015	isotig14774	4.56	4.01E-04
Pre	Nervous System	K15293	isotig08831	1.76	9.82E-05
Pre	Nervous System	K15296	isotig15948	-1.41	1.50E-05
Pre	Nervous System	K15375	isotig07308	2.89	1.77E-15
Pre	Nervous System	K16172	isotig07261	1.31	9.10E-09
Pre	Pancreatic secretion	K01539	isotig13411	-1.51	1.88E-04
Pre	Pancreatic secretion	K02677	isotig08175	1.89	4.34E-15
Pre	Pancreatic secretion	K04131	isotig15521	4.29	1.12E-05
Pre	Pancreatic secretion	K04392	isotig15866	-1.32	3.14E-05
Pre	Pancreatic secretion	K04926	isotig04414	2.12	7.18E-05
Pre	Pancreatic secretion	K04936	isotig13985	3.93	1.32E-07
Pre	Pancreatic secretion	K04962	isotig13331	2.69	9.99E-13
Pre	Pancreatic secretion	K05858	isotig03047	1.1	2.44E-07
Pre	Pancreatic secretion	K07904	isotig16757	-1.93	6.15E-07
Pre	Pancreatic secretion	K07904	isotig16854	-2.47	1.27E-13
Pre	Pancreatic secretion	K08049	isotig02947	2	3.38E-21

Pre	Retrograde endocannabinoid signaling	K02677	isotig08175	1.89	4.34E-15
Pre	Retrograde endocannabinoid signaling	K04345	isotig02324	1.78	2.56E-20
Pre	Retrograde endocannabinoid signaling	K04345	isotig08250	-1.94	3.80E-12
Pre	Retrograde endocannabinoid signaling	K04440	isotig14539	-1.19	1.17E-05
Pre	Retrograde endocannabinoid signaling	K04539	isotig08600	2.97	2.65E-05
Pre	Retrograde endocannabinoid signaling	K05858	isotig03047	1.1	2.44E-07
Pre	Retrograde endocannabinoid signaling	K08049	isotig02947	2	3.38E-21
Pre	Retrograde endocannabinoid signaling	K12302	isotig17247	4.14	3.35E-05
Pre	Retrograde endocannabinoid signaling	K15015	isotig14774	4.56	4.01E-04
Pre	Salivary secretion	K01539	isotig13411	-1.51	1.88E-04
Pre	Salivary secretion	K02183	isotig10702	3.67	5.23E-04
Pre	Salivary secretion	K02677	isotig08175	1.89	4.34E-15
Pre	Salivary secretion	K04131	isotig15521	4.29	1.12E-05
Pre	Salivary secretion	K04345	isotig02324	1.78	2.56E-20
Pre	Salivary secretion	K04345	isotig08250	-1.94	3.80E-12
Pre	Salivary secretion	K04936	isotig13985	3.93	1.32E-07
Pre	Salivary secretion	K05858	isotig03047	1.1	2.44E-07
Pre	Salivary secretion	K08049	isotig02947	2	3.38E-21
Pre	Salivary secretion	K12319	isotig15721	2.97	1.54E-06
Pre	Sensory System	K02183	isotig10702	3.67	5.23E-04
Pre	Sensory System	K02677	isotig08175	1.89	4.34E-15
Pre	Sensory System	K04345	isotig02324	1.78	2.56E-20
Pre	Sensory System	K04345	isotig08250	-1.94	3.80E-12

Pre	Sensory System	K05692	isotig15184	-1.32	1.43E-32
Pre	Sensory System	K05692	isotig06868	4.77	0.00E+00
Pre	Sensory System	K05858	isotig03047	1.1	2.44E-07
Pre	Signal Transduction	K00412	isotig12250	4.56	4.01E-04
Pre	Signal Transduction	K00626	isotig14729	-2.19	1.20E-20
Pre	Signal Transduction	K00889	isotig10052	-1.84	3.22E-07
Pre	Signal Transduction	K00901	isotig05537	2.74	2.32E-17
Pre	Signal Transduction	K01047	isotig04965	1.17	4.69E-04
Pre	Signal Transduction	K01099	isotig09832	2.39	1.85E-07
Pre	Signal Transduction	K01915	isotig11444	-2.66	5.78E-06
Pre	Signal Transduction	K02105	isotig13760	-1.19	4.38E-06
Pre	Signal Transduction	K02183	isotig10702	3.67	5.23E-04
Pre	Signal Transduction	K02599	isotig14622	-3.94	1.42E-04
Pre	Signal Transduction	K02677	isotig08175	1.89	4.34E-15
Pre	Signal Transduction	K02991	isotig19150	4.67	2.33E-04
Pre	Signal Transduction	K03097	isotig08256	1.33	1.50E-11
Pre	Signal Transduction	K03099	isotig08975	2.25	6.33E-22
Pre	Signal Transduction	K03259	isotig11110	-1.23	8.67E-06
Pre	Signal Transduction	K03868	isotig19452	-2.43	2.64E-06
Pre	Signal Transduction	K04131	isotig15521	4.29	1.12E-05
Pre	Signal Transduction	K04163	isotig18466	4.43	6.94E-04
Pre	Signal Transduction	K04345	isotig02324	1.78	2.56E-20
Pre	Signal Transduction	K04345	isotig08250	-1.94	3.80E-12
Pre	Signal Transduction	K04348	isotig05039	3.1	1.80E-14
Pre	Signal Transduction	K04358	isotig11638	3.24	1.87E-06
Pre	Signal Transduction	K04364	isotig17494	-1.57	6.90E-04
Pre	Signal Transduction	K04368	isotig05365	1.42	9.34E-05
Pre	Signal Transduction	K04375	isotig13308	-1.03	4.75E-04
Pre	Signal Transduction	K04392	isotig15866	-1.32	3.14E-05
Pre	Signal Transduction	K04440	isotig14539	-1.19	1.17E-05
Pre	Signal Transduction	K04446	isotig07756	-1.68	8.36E-07
Pre	Signal Transduction	K04459	isotig16388	5.14	1.78E-09

Pre	Signal Transduction	K04459	isotig15433	1.81	7.06E-06
Pre	Signal Transduction	K04463	isotig14730	1.78	6.09E-04
Pre	Signal Transduction	K04500	isotig07964	2.08	1.80E-11
Pre	Signal Transduction	K04501	isotig13571	-3.06	4.46E-15
Pre	Signal Transduction	K04505	isotig03446	1.03	3.58E-08
Pre	Signal Transduction	K04512	isotig13510	-3.84	4.77E-07
Pre	Signal Transduction	K04674	isotig14825	-1.92	4.30E-05
Pre	Signal Transduction	K04962	isotig13331	2.69	9.99E-13
Pre	Signal Transduction	K05315	isotig14666	3.73	1.21E-06
Pre	Signal Transduction	K05316	isotig05441	4	3.44E-21
Pre	Signal Transduction	K05327	isotig13387	1.71	5.66E-04
Pre	Signal Transduction	K05760	isotig11130	2.03	1.36E-06
Pre	Signal Transduction	K05858	isotig03047	1.1	2.44E-07
Pre	Signal Transduction	K05860	isotig13702	2.78	1.24E-04
Pre	Signal Transduction	K05863	isotig16228	-5.35	1.31E-10
Pre	Signal Transduction	K05863	isotig16053	2.25	1.56E-116
Pre	Signal Transduction	K05863	isotig16273	4.71	1.20E-48
Pre	Signal Transduction	K06058	isotig09500	-1.86	4.86E-08
Pre	Signal Transduction	K06171	isotig13855	-1.14	7.95E-06
Pre	Signal Transduction	K06619	isotig13791	1.56	1.66E-04
Pre	Signal Transduction	K07293	isotig01787	1.71	3.33E-24
Pre	Signal Transduction	K07298	isotig14684	-1.61	9.99E-05
Pre	Signal Transduction	K08049	isotig02947	2	3.38E-21
Pre	Signal Transduction	K08960	isotig15816	-2.39	5.20E-10
Pre	Signal Transduction	K10151	isotig16594	-3.61	7.60E-04
Pre	Signal Transduction	K12646	isotig06081	-1.53	1.81E-12
Pre	Signal Transduction	K13711	isotig14389	-2.06	2.82E-06
Pre	Signal Transduction	K13711	isotig05661	-1.33	5.95E-04
Pre	Signal Transduction	K13755	isotig08310	4.29	1.12E-05
Pre	Signal Transduction	K15759	isotig09342	1.76	7.10E-08
Pre	Signal Transduction	K16056	isotig10128	-1.61	1.66E-04
Pre	Substance Dependence	K01120	isotig08927	3.08	9.95E-13

Pre	Substance Dependence	K01120	isotig11262	3.56	6.18E-06
Pre	Substance Dependence	K02183	isotig10702	3.67	5.23E-04
Pre	Substance Dependence	K02677	isotig08175	1.89	4.34E-15
Pre	Substance Dependence	K03099	isotig08975	2.25	6.33E-22
Pre	Substance Dependence	K04345	isotig02324	1.78	2.56E-20
Pre	Substance Dependence	K04345	isotig08250	-1.94	3.80E-12
Pre	Substance Dependence	K04348	isotig05039	3.1	1.80E-14
Pre	Substance Dependence	K04364	isotig17494	-1.57	6.90E-04
Pre	Substance Dependence	K04368	isotig05365	1.42	9.34E-05
Pre	Substance Dependence	K04539	isotig08600	2.97	2.65E-05
Pre	Substance Dependence	K04630	isotig08244	-1.29	4.52E-07
Pre	Substance Dependence	K07359	isotig12228	3.88	1.74E-04
Pre	Substance Dependence	K08049	isotig02947	2	3.38E-21
Pre	Substance Dependence	K08155	isotig10076	3.97	5.09E-11
Pre	Substance Dependence	K11252	isotig19182	4.97	4.65E-05
Pre	Substance Dependence	K11253	isotig19439	-4.73	1.99E-04
Pre	Substance Dependence	K11253	isotig19510	-4.28	1.52E-05
Pre	Substance Dependence	K11254	isotig16986	1.4	1.34E-04
Pre	Substance Dependence	K12302	isotig17247	4.14	3.35E-05
Pre	Substance Dependence	K13296	isotig04542	2.45	2.79E-25
Pre	Substance Dependence	K13755	isotig08310	4.29	1.12E-05
Pre	Substance Dependence	K15015	isotig14774	4.56	4.01E-04
Pre	Synaptic vesicle cycle	K01528	isotig02826	1.27	4.00E-18
Pre	Synaptic vesicle cycle	K02152	isotig19022	3.67	2.07E-06
Pre	Synaptic vesicle cycle	K04646	isotig13231	-1.75	5.92E-26
Pre	Synaptic vesicle cycle	K07882	isotig15140	3.85	4.82E-10
Pre	Synaptic vesicle cycle	K08155	isotig10076	3.97	5.09E-11
Pre	Synaptic vesicle cycle	K12302	isotig17247	4.14	3.35E-05
Pre	Synaptic vesicle cycle	K14636	isotig14851	4.06	5.82E-05
Pre	Synaptic vesicle cycle	K15015	isotig14774	4.56	4.01E-04
Pre	Synaptic vesicle cycle	K15293	isotig08831	1.76	9.82E-05
Pre	Vascular smooth muscle	K02183	isotig10702	3.67	5.23E-04

	contraction				
Pre	Vascular smooth muscle contraction	K02677	isotig08175	1.89	4.34E-15
Pre	Vascular smooth muscle contraction	K04345	isotig02324	1.78	2.56E-20
Pre	Vascular smooth muscle contraction	K04345	isotig08250	-1.94	3.80E-12
Pre	Vascular smooth muscle contraction	K04368	isotig05365	1.42	9.34E-05
Pre	Vascular smooth muscle contraction	K04936	isotig13985	3.93	1.32E-07
Pre	Vascular smooth muscle contraction	K05858	isotig03047	1.1	2.44E-07
Pre	Vascular smooth muscle contraction	K06068	isotig04374	1.73	1.66E-11
Pre	Vascular smooth muscle contraction	K06068	isotig03922	1.25	5.41E-07
Pre	Vascular smooth muscle contraction	K08049	isotig02947	2	3.38E-21
Pre	Vascular smooth muscle contraction	K12319	isotig15721	2.97	1.54E-06
Pre	Vascular smooth muscle contraction	K12323	isotig08178	1.53	3.89E-08
Post	Amino Acid Metabolism	K00022	isotig15495	-4.06	1.94E-70
Post	Amino Acid Metabolism	K00128	isotig09962	-2.22	9.34E-15
Post	Amino Acid Metabolism	K00129	isotig14559	-4.27	2.26E-58
Post	Amino Acid Metabolism	K00143	isotig06177	-1.01	4.91E-05
Post	Amino Acid Metabolism	K00162	isotig15346	-2.46	4.42E-05
Post	Amino Acid Metabolism	K00252	isotig06497	-1.64	1.47E-09
Post	Amino Acid Metabolism	K00253	isotig10260	-1.85	4.00E-05
Post	Amino Acid Metabolism	K00264	isotig04549	1.09	1.33E-11
Post	Amino Acid Metabolism	K00272	isotig04789	1.86	6.37E-04

Post	Amino Acid Metabolism	K00281	isotig14245	-3.03	1.88E-05
Post	Amino Acid Metabolism	K00286	isotig01155	1.56	5.23E-23
Post	Amino Acid Metabolism	K00382	isotig14339	-2.11	3.92E-18
Post	Amino Acid Metabolism	K00457	isotig09530	-3.84	4.68E-21
Post	Amino Acid Metabolism	K00472	isotig09722	-2.06	5.41E-19
Post	Amino Acid Metabolism	K00472	isotig14476	-7.05	8.02E-16
Post	Amino Acid Metabolism	K00472	isotig14283	-5.42	2.60E-06
Post	Amino Acid Metabolism	K00493	isotig14601	-4.94	6.67E-05
Post	Amino Acid Metabolism	K00505	isotig07711	-6.06	4.86E-202
Post	Amino Acid Metabolism	K00505	isotig13471	-2.12	1.62E-08
Post	Amino Acid Metabolism	K00549	isotig13593	-3.57	1.05E-14
Post	Amino Acid Metabolism	K00599	isotig16397	-2.41	7.18E-05
Post	Amino Acid Metabolism	K00599	isotig11948	-1.99	4.55E-05
Post	Amino Acid Metabolism	K00600	isotig11198	-1.7	8.13E-10
Post	Amino Acid Metabolism	K00626	isotig14729	-2.19	1.20E-20
Post	Amino Acid Metabolism	K00657	isotig15794	-2.78	1.39E-05
Post	Amino Acid Metabolism	K00789	isotig07993	-1.95	1.04E-18
Post	Amino Acid Metabolism	K00797	isotig16616	-5.12	2.24E-05
Post	Amino Acid Metabolism	K00815	isotig13862	-3.87	1.23E-24
Post	Amino Acid Metabolism	K00820	isotig13749	-7.21	1.98E-33
Post	Amino Acid Metabolism	K00827	isotig06701	-4.86	3.13E-14
Post	Amino Acid Metabolism	K00934	isotig08028	2.09	3.47E-86
Post	Amino Acid Metabolism	K01011	isotig11596	3.73	9.06E-12
Post	Amino Acid Metabolism	K01425	isotig16061	4.78	1.36E-04
Post	Amino Acid Metabolism	K01555	isotig03152	-1.22	4.54E-07
Post	Amino Acid Metabolism	K01641	isotig14068	-1.88	1.35E-07
Post	Amino Acid Metabolism	K01697	isotig09350	-1.21	1.01E-05
Post	Amino Acid Metabolism	K01697	isotig13714	-1.79	2.46E-08
Post	Amino Acid Metabolism	K01738	isotig16370	-2.03	1.74E-06
Post	Amino Acid Metabolism	K01758	isotig10842	-3.06	4.56E-19
Post	Amino Acid Metabolism	K01915	isotig11444	-2.66	5.78E-06
Post	Amino Acid Metabolism	K01953	isotig14208	-1.87	7.04E-13

Post	Amino Acid Metabolism	K06101	isotig13232	1.84	1.47E-19
Post	Amino Acid Metabolism	K07508	isotig11546	1.16	1.71E-09
Post	Amino Acid Metabolism	K08660	isotig14577	-2.58	9.06E-18
Post	Amino Acid Metabolism	K09699	isotig11326	-2.39	4.31E-06
Post	Amino Acid Metabolism	K09880	isotig08715	2.63	3.42E-04
Post	Amino Acid Metabolism	K11419	isotig09704	-1.7	2.09E-04
Post	Amino Acid Metabolism	K11420	isotig08337	-1.36	5.88E-07
Post	Amino Acid Metabolism	K11424	isotig13821	-3.73	4.61E-09
Post	Amino Acid Metabolism	K11538	isotig15134	-3	8.97E-17
Post	Amino Acid Metabolism	K13647	isotig09138	-1.97	2.30E-08
Post	Amino Acid Metabolism	K13953	isotig17766	5.78	8.58E-08
Post	Amino Acid Metabolism	K14455	isotig14718	-1.24	7.24E-05
Post	Amino Acid Metabolism	K15633	isotig14097	-1.14	4.18E-05
Post	Amino sugar and nucleotide sugar metabolism	K00012	isotig06369	1.06	2.97E-04
Post	Amino sugar and nucleotide sugar metabolism	K00621	isotig18963	-4.73	1.99E-04
Post	Amino sugar and nucleotide sugar metabolism	K00698	isotig09218	-4.73	1.44E-30
Post	Amino sugar and nucleotide sugar metabolism	K00820	isotig13749	-7.21	1.98E-33
Post	Amino sugar and nucleotide sugar metabolism	K00844	isotig14064	-2.21	2.76E-07
Post	Amino sugar and nucleotide sugar metabolism	K00966	isotig09908	-2.53	1.04E-10
Post	Amino sugar and nucleotide sugar metabolism	K00966	isotig14721	-1.71	3.02E-05
Post	Amino sugar and nucleotide sugar metabolism	K01183	isotig14312	-2.53	7.16E-04
Post	Amino sugar and nucleotide sugar metabolism	K01784	isotig15366	-1.87	1.90E-08
Post	Amino sugar and nucleotide	K01835	isotig05893	1.1	8.67E-06

	sugar metabolism				
Post	Amino sugar and nucleotide sugar metabolism	K01836	isotig09654	-1.76	1.86E-09
Post	Amino sugar and nucleotide sugar metabolism	K01840	isotig16968	-3.2	1.22E-04
Post	Amino sugar and nucleotide sugar metabolism	K02377	isotig15639	-1.72	5.88E-04
Post	Amino sugar and nucleotide sugar metabolism	K05305	isotig09448	-3.47	1.49E-62
Post	Amino sugar and nucleotide sugar metabolism	K12373	isotig14029	-2.98	3.35E-15
Post	Carbohydrate Metabolism	K00012	isotig06369	1.06	2.97E-04
Post	Carbohydrate Metabolism	K00022	isotig15495	-4.06	1.94E-70
Post	Carbohydrate Metabolism	K00026	isotig15407	-1.49	1.64E-04
Post	Carbohydrate Metabolism	K00030	isotig14644	1.13	1.95E-04
Post	Carbohydrate Metabolism	K00030	isotig15956	-1.27	5.21E-05
Post	Carbohydrate Metabolism	K00033	isotig14147	-1.46	7.53E-09
Post	Carbohydrate Metabolism	K00045	isotig14267	-7.6	1.10E-21
Post	Carbohydrate Metabolism	K00128	isotig09962	-2.22	9.34E-15
Post	Carbohydrate Metabolism	K00129	isotig14559	-4.27	2.26E-58
Post	Carbohydrate Metabolism	K00162	isotig15346	-2.46	4.42E-05
Post	Carbohydrate Metabolism	K00382	isotig14339	-2.11	3.92E-18
Post	Carbohydrate Metabolism	K00600	isotig11198	-1.7	8.13E-10
Post	Carbohydrate Metabolism	K00615	isotig13839	-1.9	2.66E-17
Post	Carbohydrate Metabolism	K00621	isotig18963	-4.73	1.99E-04
Post	Carbohydrate Metabolism	K00626	isotig14729	-2.19	1.20E-20
Post	Carbohydrate Metabolism	K00627	isotig13822	-1.15	8.08E-05
Post	Carbohydrate Metabolism	K00693	isotig09718	-1.12	6.44E-06
Post	Carbohydrate Metabolism	K00698	isotig09218	-4.73	1.44E-30
Post	Carbohydrate Metabolism	K00699	isotig05471	2.23	9.48E-10
Post	Carbohydrate Metabolism	K00820	isotig13749	-7.21	1.98E-33
Post	Carbohydrate Metabolism	K00844	isotig14064	-2.21	2.76E-07

Post	Carbohydrate Metabolism	K00850	isotig09210	-1.95	2.75E-18
Post	Carbohydrate Metabolism	K00873	isotig14289	-2.48	1.99E-20
Post	Carbohydrate Metabolism	K00889	isotig10052	-1.84	3.22E-07
Post	Carbohydrate Metabolism	K00966	isotig09908	-2.53	1.04E-10
Post	Carbohydrate Metabolism	K00966	isotig14721	-1.71	3.02E-05
Post	Carbohydrate Metabolism	K01099	isotig09832	2.39	1.85E-07
Post	Carbohydrate Metabolism	K01103	isotig06209	1.33	4.40E-05
Post	Carbohydrate Metabolism	K01179	isotig14720	7.19	1.05E-33
Post	Carbohydrate Metabolism	K01183	isotig14312	-2.53	7.16E-04
Post	Carbohydrate Metabolism	K01194	isotig13930	-4.21	1.49E-24
Post	Carbohydrate Metabolism	K01194	isotig14140	-3.42	2.36E-05
Post	Carbohydrate Metabolism	K01610	isotig16257	4.56	4.01E-04
Post	Carbohydrate Metabolism	K01637	isotig14870	4.56	4.01E-04
Post	Carbohydrate Metabolism	K01638	isotig10314	6.53	6.79E-12
Post	Carbohydrate Metabolism	K01641	isotig14068	-1.88	1.35E-07
Post	Carbohydrate Metabolism	K01648	isotig13341	-2.68	1.33E-51
Post	Carbohydrate Metabolism	K01689	isotig14669	-1.15	4.53E-17
Post	Carbohydrate Metabolism	K01784	isotig15366	-1.87	1.90E-08
Post	Carbohydrate Metabolism	K01835	isotig05893	1.1	8.67E-06
Post	Carbohydrate Metabolism	K01836	isotig09654	-1.76	1.86E-09
Post	Carbohydrate Metabolism	K01840	isotig16968	-3.2	1.22E-04
Post	Carbohydrate Metabolism	K01895	isotig09700	6.1	2.49E-09
Post	Carbohydrate Metabolism	K01899	isotig15371	1.14	4.48E-05
Post	Carbohydrate Metabolism	K01900	isotig14845	-1.14	5.80E-04
Post	Carbohydrate Metabolism	K01915	isotig11444	-2.66	5.78E-06
Post	Carbohydrate Metabolism	K02377	isotig15639	-1.72	5.88E-04
Post	Carbohydrate Metabolism	K03841	isotig05958	-1.3	1.25E-11
Post	Carbohydrate Metabolism	K05305	isotig09448	-3.47	1.49E-62
Post	Carbohydrate Metabolism	K05858	isotig03047	1.1	2.44E-07
Post	Carbohydrate Metabolism	K05860	isotig13702	2.78	1.24E-04
Post	Carbohydrate Metabolism	K11262	isotig03641	-2.82	1.28E-33
Post	Carbohydrate Metabolism	K12309	isotig10126	-4.42	4.94E-06

Post	Carbohydrate Metabolism	K12373	isotig14029	-2.98	3.35E-15
Post	Carbohydrate Metabolism	K13711	isotig14389	-2.06	2.82E-06
Post	Carbohydrate Metabolism	K13711	isotig05661	-1.33	5.95E-04
Post	Carbohydrate Metabolism	K13953	isotig17766	5.78	8.58E-08
Post	Carbohydrate Metabolism	K15633	isotig14097	-1.14	4.18E-05
Post	Carbohydrate Metabolism	K15759	isotig09342	1.76	7.10E-08
Post	Cell cycle	K02087	isotig15766	-2.67	3.92E-05
Post	Cell cycle	K02180	isotig10474	-2.16	1.86E-05
Post	Cell cycle	K02209	isotig13773	-4.16	8.96E-09
Post	Cell cycle	K02210	isotig09692	-1.78	9.18E-05
Post	Cell cycle	K02540	isotig05633	-1.53	2.36E-10
Post	Cell cycle	K02541	isotig13735	-1.12	4.03E-05
Post	Cell cycle	K02542	isotig13631	-3.28	3.68E-08
Post	Cell cycle	K03358	isotig12128	-2.73	2.34E-05
Post	Cell cycle	K03364	isotig14041	-1.09	9.91E-06
Post	Cell cycle	K03868	isotig19452	-2.43	2.64E-06
Post	Cell cycle	K03875	isotig07946	-3.35	4.12E-05
Post	Cell cycle	K04500	isotig07964	2.08	1.80E-11
Post	Cell cycle	K04501	isotig13571	-3.06	4.46E-15
Post	Cell cycle	K04802	isotig04819	-1.64	3.72E-11
Post	Cell cycle	K05868	isotig15274	-4.45	9.32E-25
Post	Cell cycle	K06619	isotig13791	1.56	1.66E-04
Post	Cell cycle	K06626	isotig05737	-5.35	4.43E-06
Post	Cell cycle	K06627	isotig13667	-3.49	1.29E-09
Post	Cell cycle	K06628	isotig10620	-1.53	6.07E-04
Post	Cell cycle	K06631	isotig09584	-2.84	5.45E-09
Post	Cell cycle	K06633	isotig14206	-3.95	5.31E-20
Post	Cell cycle	K06634	isotig15993	-1.54	2.15E-04
Post	Cell cycle	K06670	isotig14224	-2.03	2.88E-05
Post	Cell cycle	K10151	isotig16594	-3.61	7.60E-04
Post	Cell Growth and Death	K01011	isotig11596	3.73	9.06E-12
Post	Cell Growth and Death	K01230	isotig05069	-2.11	1.72E-07

Post	Cell Growth and Death	K01689	isotig14669	-1.15	4.53E-17
Post	Cell Growth and Death	K01869	isotig09194	1.47	8.98E-04
Post	Cell Growth and Death	K01870	isotig13419	-1.38	5.72E-06
Post	Cell Growth and Death	K01874	isotig13483	-1.17	1.35E-04
Post	Cell Growth and Death	K01875	isotig14848	-1.71	1.56E-06
Post	Cell Growth and Death	K01876	isotig01106	1.12	3.89E-07
Post	Cell Growth and Death	K01885	isotig09784	-1.27	1.20E-05
Post	Cell Growth and Death	K01889	isotig06673	1.57	1.26E-08
Post	Cell Growth and Death	K02209	isotig13773	-4.16	8.96E-09
Post	Cell Growth and Death	K02210	isotig09692	-1.78	9.18E-05
Post	Cell Growth and Death	K02320	isotig07358	-1.25	6.22E-06
Post	Cell Growth and Death	K02321	isotig05059	1.28	5.17E-08
Post	Cell Growth and Death	K02324	isotig14972	-3.94	1.42E-04
Post	Cell Growth and Death	K02540	isotig05633	-1.53	2.36E-10
Post	Cell Growth and Death	K02541	isotig13735	-1.12	4.03E-05
Post	Cell Growth and Death	K02542	isotig13631	-3.28	3.68E-08
Post	Cell Growth and Death	K02725	isotig12414	-1.46	2.48E-10
Post	Cell Growth and Death	K02726	isotig17342	-2.27	5.67E-10
Post	Cell Growth and Death	K02737	isotig16429	-1.08	7.42E-04
Post	Cell Growth and Death	K02738	isotig16918	-1.38	1.31E-04
Post	Cell Growth and Death	K02739	isotig12252	1.59	9.51E-08
Post	Cell Growth and Death	K02866	isotig17899	1.57	1.17E-12
Post	Cell Growth and Death	K02870	isotig19382	-1.73	9.76E-04
Post	Cell Growth and Death	K02887	isotig16605	-2.73	2.34E-05
Post	Cell Growth and Death	K02896	isotig17810	1.31	2.01E-04
Post	Cell Growth and Death	K02897	isotig14470	-5.78	1.09E-07
Post	Cell Growth and Death	K02912	isotig19212	-1.51	2.69E-07
Post	Cell Growth and Death	K02917	isotig18131	5.26	3.55E-10
Post	Cell Growth and Death	K02934	isotig17195	1.28	1.70E-19
Post	Cell Growth and Death	K02934	isotig17388	-1.41	2.88E-05
Post	Cell Growth and Death	K02936	isotig16774	1.07	7.74E-10
Post	Cell Growth and Death	K02948	isotig14490	-2.54	5.90E-07

Post	Cell Growth and Death	K02960	isotig19406	1.63	1.25E-04
Post	Cell Growth and Death	K02974	isotig19035	-1.1	3.36E-04
Post	Cell Growth and Death	K02991	isotig19150	4.67	2.33E-04
Post	Cell Growth and Death	K02997	isotig18548	1.52	1.78E-19
Post	Cell Growth and Death	K03018	isotig13246	-1.11	6.59E-04
Post	Cell Growth and Death	K03020	isotig08137	-1.4	2.49E-05
Post	Cell Growth and Death	K03028	isotig13575	-1.55	1.14E-06
Post	Cell Growth and Death	K03033	isotig11658	-1.03	3.42E-04
Post	Cell Growth and Death	K03038	isotig15747	-1.02	7.54E-06
Post	Cell Growth and Death	K03062	contig01344	-3.95	0.00E+00
Post	Cell Growth and Death	K03097	isotig08256	1.33	1.50E-11
Post	Cell Growth and Death	K03107	isotig16912	1.06	7.83E-05
Post	Cell Growth and Death	K03130	isotig07708	1.51	9.24E-11
Post	Cell Growth and Death	K03137	isotig16590	-2.12	4.42E-07
Post	Cell Growth and Death	K03165	isotig13757	-1.61	7.69E-04
Post	Cell Growth and Death	K03178	isotig13405	-1.23	4.47E-08
Post	Cell Growth and Death	K03231	isotig15543	5.22	9.38E-06
Post	Cell Growth and Death	K03241	isotig09800	-1.08	3.11E-05
Post	Cell Growth and Death	K03247	isotig15254	-1.49	3.10E-05
Post	Cell Growth and Death	K03259	isotig11110	-1.23	8.67E-06
Post	Cell Growth and Death	K03260	isotig14306	-1.25	9.84E-04
Post	Cell Growth and Death	K03264	isotig12114	-2.33	1.72E-12
Post	Cell Growth and Death	K03265	isotig10096	-1.94	3.80E-12
Post	Cell Growth and Death	K03267	isotig15201	-1.19	1.16E-04
Post	Cell Growth and Death	K03358	isotig12128	-2.73	2.34E-05
Post	Cell Growth and Death	K03364	isotig14041	-1.09	9.91E-06
Post	Cell Growth and Death	K03514	isotig14120	-1.35	2.61E-05
Post	Cell Growth and Death	K03539	isotig16563	-2.53	7.16E-04
Post	Cell Growth and Death	K03679	isotig16142	-1.43	6.58E-04
Post	Cell Growth and Death	K03868	isotig19452	-2.43	2.64E-06
Post	Cell Growth and Death	K03875	isotig07946	-3.35	4.12E-05
Post	Cell Growth and Death	K04440	isotig14539	-1.19	1.17E-05

Post	Cell Growth and Death	K04523	isotig14054	-1.25	5.57E-12
Post	Cell Growth and Death	K04554	isotig15673	-1.08	2.22E-05
Post	Cell Growth and Death	K04567	isotig14388	-1.75	2.45E-07
Post	Cell Growth and Death	K04802	isotig04819	-1.64	3.72E-11
Post	Cell Growth and Death	K05019	isotig18485	1.86	6.37E-04
Post	Cell Growth and Death	K05546	isotig09021	-1.64	6.32E-11
Post	Cell Growth and Death	K06634	isotig15993	-1.54	2.15E-04
Post	Cell Growth and Death	K06943	isotig09466	1.89	5.48E-66
Post	Cell Growth and Death	K07151	isotig13389	-1.45	6.49E-09
Post	Cell Growth and Death	K07151	isotig09144	-2	1.29E-14
Post	Cell Growth and Death	K07562	isotig14040	-1.44	1.21E-05
Post	Cell Growth and Death	K08489	isotig16027	-1.7	2.09E-04
Post	Cell Growth and Death	K08490	isotig14840	-1.93	2.19E-08
Post	Cell Growth and Death	K08495	isotig15031	1.23	6.15E-04
Post	Cell Growth and Death	K08509	isotig15489	-1.13	2.94E-04
Post	Cell Growth and Death	K08517	isotig16798	-2.77	1.82E-07
Post	Cell Growth and Death	K08734	isotig06477	-1.74	1.73E-06
Post	Cell Growth and Death	K09485	isotig14420	-1.18	1.16E-07
Post	Cell Growth and Death	K09486	isotig13892	-1.4	3.55E-10
Post	Cell Growth and Death	K09523	isotig07699	-1.26	9.62E-11
Post	Cell Growth and Death	K09540	isotig13550	-1.87	8.11E-08
Post	Cell Growth and Death	K09561	isotig08739	-1.16	3.63E-05
Post	Cell Growth and Death	K09580	isotig14036	-1.39	5.53E-48
Post	Cell Growth and Death	K10080	isotig14296	-2.31	2.80E-06
Post	Cell Growth and Death	K10573	isotig18694	-2.28	7.64E-04
Post	Cell Growth and Death	K10582	isotig15108	-2.3	6.44E-08
Post	Cell Growth and Death	K10586	isotig13420	-1.69	1.18E-07
Post	Cell Growth and Death	K10592	isotig13237	1.34	8.90E-16
Post	Cell Growth and Death	K10643	isotig02131	3.03	1.24E-143
Post	Cell Growth and Death	K10685	isotig14182	-1.03	1.22E-04
Post	Cell Growth and Death	K10756	isotig15750	-1.51	1.88E-04
Post	Cell Growth and Death	K10773	isotig12166	-2.13	7.55E-05

Post	Cell Growth and Death	K10846	isotig09168	1.25	1.30E-11
Post	Cell Growth and Death	K10875	isotig09192	-4.46	9.26E-11
Post	Cell Growth and Death	K11583	isotig05144	1.21	6.68E-07
Post	Cell Growth and Death	K11718	isotig13229	-1.59	2.44E-06
Post	Cell Growth and Death	K12172	isotig14246	-1.25	7.34E-06
Post	Cell Growth and Death	K12275	isotig14521	-1.68	3.77E-20
Post	Cell Growth and Death	K12584	isotig17252	-2.38	8.90E-14
Post	Cell Growth and Death	K12591	isotig09200	1.43	3.13E-12
Post	Cell Growth and Death	K12613	isotig09492	-1.78	1.33E-05
Post	Cell Growth and Death	K12614	isotig14198	-1.03	9.48E-06
Post	Cell Growth and Death	K12666	isotig10230	-1.17	1.61E-14
Post	Cell Growth and Death	K12820	isotig13798	-1.1	2.82E-04
Post	Cell Growth and Death	K12823	isotig08511	1.94	1.53E-60
Post	Cell Growth and Death	K12831	isotig14849	-2.64	1.86E-08
Post	Cell Growth and Death	K12838	isotig05019	1.1	6.42E-06
Post	Cell Growth and Death	K12840	isotig16867	-1.23	7.02E-04
Post	Cell Growth and Death	K12858	isotig09428	-1.03	4.75E-04
Post	Cell Growth and Death	K12863	isotig16247	1.05	2.19E-04
Post	Cell Growth and Death	K12867	isotig13513	-1.11	1.06E-04
Post	Cell Growth and Death	K12872	isotig14883	-1.35	1.88E-06
Post	Cell Growth and Death	K12881	isotig14940	-1.12	3.96E-07
Post	Cell Growth and Death	K12891	isotig06924	1.74	4.21E-07
Post	Cell Growth and Death	K13114	isotig04729	2.08	2.82E-62
Post	Cell Growth and Death	K13126	isotig13759	1.21	9.82E-78
Post	Cell Growth and Death	K13126	isotig14448	-3.58	2.40E-19
Post	Cell Growth and Death	K13130	isotig16834	-2.57	2.41E-06
Post	Cell Growth and Death	K13151	isotig15468	-1.33	7.07E-05
Post	Cell Growth and Death	K13250	isotig17616	-1.24	2.71E-05
Post	Cell Growth and Death	K13251	isotig17940	-2.69	5.19E-07
Post	Cell Growth and Death	K13431	isotig14019	-1.08	7.42E-04
Post	Cell Growth and Death	K13917	isotig06525	1.6	2.11E-14
Post	Cell Growth and Death	K13993	isotig17002	-4.94	3.26E-08

Post	Cell Growth and Death	K14006	isotig13564	-1.43	5.04E-08
Post	Cell Growth and Death	K14007	isotig13426	-1.4	7.76E-06
Post	Cell Growth and Death	K14010	contig01758	-4.59	0.00E+00
Post	Cell Growth and Death	K14026	isotig13805	-1.48	5.99E-09
Post	Cell Growth and Death	K14168	isotig15251	1.13	1.36E-05
Post	Cell Growth and Death	K14283	isotig15286	-1.18	2.22E-04
Post	Cell Growth and Death	K14291	isotig15059	-1.85	1.25E-07
Post	Cell Growth and Death	K14292	isotig14910	-1.84	1.00E-04
Post	Cell Growth and Death	K14294	isotig17147	-2.21	2.76E-07
Post	Cell Growth and Death	K14298	isotig15713	-1.58	5.30E-05
Post	Cell Growth and Death	K14299	isotig15623	-1.61	7.69E-04
Post	Cell Growth and Death	K14315	isotig06669	-1.12	4.74E-05
Post	Cell Growth and Death	K14317	isotig13325	-1.01	1.53E-06
Post	Cell Growth and Death	K14406	isotig06585	-2.03	7.37E-05
Post	Cell Growth and Death	K14408	isotig13783	1.07	1.59E-08
Post	Cell Growth and Death	K14416	isotig10884	-1.56	6.75E-07
Post	Cell Growth and Death	K14537	isotig13790	-1.3	1.42E-06
Post	Cell Growth and Death	K14539	isotig06125	-2.36	2.28E-28
Post	Cell Growth and Death	K14546	isotig16786	-1.73	9.76E-04
Post	Cell Growth and Death	K14553	isotig11710	1.17	8.65E-06
Post	Cell Growth and Death	K14561	isotig15848	1.77	7.58E-09
Post	Cell Growth and Death	K14650	isotig13339	1.11	5.98E-10
Post	Cell Growth and Death	K14962	isotig16322	-2.98	1.68E-06
Post	Cell Growth and Death	K15363	isotig03001	1.46	7.68E-18
Post	Cell Growth and Death	K15544	isotig12456	-2.66	5.78E-06
Post	Fructose and mannose metabolism	K00045	isotig14267	-7.6	1.10E-21
Post	Fructose and mannose metabolism	K00850	isotig09210	-1.95	2.75E-18
Post	Fructose and mannose metabolism	K00966	isotig09908	-2.53	1.04E-10
Post	Fructose and mannose	K00966	isotig14721	-1.71	3.02E-05

	metabolism				
Post	Fructose and mannose metabolism	K01103	isotig06209	1.33	4.40E-05
Post	Fructose and mannose metabolism	K01840	isotig16968	-3.2	1.22E-04
Post	Fructose and mannose metabolism	K02377	isotig15639	-1.72	5.88E-04
Post	Fructose and mannose metabolism	K03841	isotig05958	-1.3	1.25E-11
Post	Fructose and mannose metabolism	K05305	isotig09448	-3.47	1.49E-62
Post	Genetic information processing	K02087	isotig15766	-2.67	3.92E-05
Post	Genetic information processing	K02180	isotig10474	-2.16	1.86E-05
Post	Genetic information processing	K02183	isotig10702	3.67	5.23E-04
Post	Genetic information processing	K02209	isotig13773	-4.16	8.96E-09
Post	Genetic information processing	K02210	isotig09692	-1.78	9.18E-05
Post	Genetic information processing	K02540	isotig05633	-1.53	2.36E-10
Post	Genetic information processing	K02541	isotig13735	-1.12	4.03E-05
Post	Genetic information processing	K02542	isotig13631	-3.28	3.68E-08
Post	Genetic information processing	K03358	isotig12128	-2.73	2.34E-05
Post	Genetic information processing	K03364	isotig14041	-1.09	9.91E-06
Post	Genetic information	K03868	isotig19452	-2.43	2.64E-06

	processing				
Post	Genetic information processing	K03875	isotig07946	-3.35	4.12E-05
Post	Genetic information processing	K04345	isotig02324	1.78	2.56E-20
Post	Genetic information processing	K04345	isotig08250	-1.94	3.80E-12
Post	Genetic information processing	K04348	isotig05039	3.1	1.80E-14
Post	Genetic information processing	K04368	isotig05365	1.42	9.34E-05
Post	Genetic information processing	K04500	isotig07964	2.08	1.80E-11
Post	Genetic information processing	K04501	isotig13571	-3.06	4.46E-15
Post	Genetic information processing	K04739	isotig06637	2.11	6.92E-39
Post	Genetic information processing	K04802	isotig04819	-1.64	3.72E-11
Post	Genetic information processing	K05868	isotig15963	-5.84	6.44E-08
Post	Genetic information processing	K05868	isotig15274	-4.45	9.32E-25
Post	Genetic information processing	K06619	isotig13791	1.56	1.66E-04
Post	Genetic information processing	K06626	isotig05737	-5.35	4.43E-06
Post	Genetic information processing	K06627	isotig13667	-3.49	1.29E-09
Post	Genetic information processing	K06628	isotig10620	-1.53	6.07E-04
Post	Genetic information	K06631	isotig09584	-2.84	5.45E-09

	processing				
Post	Genetic information processing	K06633	isotig14206	-3.95	5.31E-20
Post	Genetic information processing	K06634	isotig15993	-1.54	2.15E-04
Post	Genetic information processing	K06670	isotig14224	-2.03	2.88E-05
Post	Genetic information processing	K08049	isotig02947	2	3.38E-21
Post	Genetic information processing	K09175	isotig08355	3.78	3.02E-04
Post	Genetic information processing	K10151	isotig16594	-3.61	7.60E-04
Post	Genetic information processing	K11481	isotig16081	-4.78	1.74E-07
Post	Genetic information processing	K11481	isotig16435	-2.4	1.76E-05
Post	Glycolysis / Gluconeogenesis	K00128	isotig09962	-2.22	9.34E-15
Post	Glycolysis / Gluconeogenesis	K00129	isotig14559	-4.27	2.26E-58
Post	Glycolysis / Gluconeogenesis	K00162	isotig15346	-2.46	4.42E-05
Post	Glycolysis / Gluconeogenesis	K00627	isotig13822	-1.15	8.08E-05
Post	Glycolysis / Gluconeogenesis	K00844	isotig14064	-2.21	2.76E-07
Post	Glycolysis / Gluconeogenesis	K00850	isotig09210	-1.95	2.75E-18
Post	Glycolysis / Gluconeogenesis	K00873	isotig14289	-2.48	1.99E-20
Post	Glycolysis / Gluconeogenesis	K01610	isotig16257	4.56	4.01E-04
Post	Glycolysis / Gluconeogenesis	K01689	isotig14669	-1.15	4.53E-17
Post	Glycolysis / Gluconeogenesis	K01835	isotig05893	1.1	8.67E-06
Post	Glycolysis / Gluconeogenesis	K01895	isotig09700	6.1	2.49E-09
Post	Glycolysis / Gluconeogenesis	K03841	isotig05958	-1.3	1.25E-11
Post	Glycolysis / Gluconeogenesis	K13953	isotig17766	5.78	8.58E-08
Post	Glycolysis / Gluconeogenesis	K15633	isotig14097	-1.14	4.18E-05
Post	Lipid Metabolism	K00006	isotig15788	-2.05	1.68E-07

Post	Lipid Metabolism	K00022	isotig15495	-4.06	1.94E-70
Post	Lipid Metabolism	K00128	isotig09962	-2.22	9.34E-15
Post	Lipid Metabolism	K00232	isotig14004	-3.12	3.33E-07
Post	Lipid Metabolism	K00252	isotig06497	-1.64	1.47E-09
Post	Lipid Metabolism	K00432	isotig17231	4.96	5.47E-37
Post	Lipid Metabolism	K00432	isotig15233	2.1	2.62E-41
Post	Lipid Metabolism	K00493	isotig14601	-4.94	6.67E-05
Post	Lipid Metabolism	K00507	isotig15805	4.88	7.89E-05
Post	Lipid Metabolism	K00507	isotig10762	-1.44	1.59E-25
Post	Lipid Metabolism	K00626	isotig14729	-2.19	1.20E-20
Post	Lipid Metabolism	K00645	isotig05972	1.69	3.51E-04
Post	Lipid Metabolism	K00649	isotig09634	-1.95	7.04E-05
Post	Lipid Metabolism	K00650	isotig16510	-3.84	2.50E-04
Post	Lipid Metabolism	K00654	isotig10028	-1.38	2.51E-04
Post	Lipid Metabolism	K00699	isotig05471	2.23	9.48E-10
Post	Lipid Metabolism	K00864	isotig09958	-2.07	6.97E-17
Post	Lipid Metabolism	K00901	isotig05537	2.74	2.32E-17
Post	Lipid Metabolism	K00993	isotig15000	-2.2	1.20E-04
Post	Lipid Metabolism	K01047	isotig04965	1.17	4.69E-04
Post	Lipid Metabolism	K01062	isotig14457	1.6	2.46E-13
Post	Lipid Metabolism	K01062	isotig09412	-1.37	9.48E-06
Post	Lipid Metabolism	K01074	isotig04510	-1.35	9.76E-07
Post	Lipid Metabolism	K01080	isotig15255	-4.08	1.06E-18
Post	Lipid Metabolism	K01115	isotig13270	-1.56	1.95E-09
Post	Lipid Metabolism	K01201	isotig14368	-3.42	2.36E-05
Post	Lipid Metabolism	K01201	isotig10400	-2.41	3.27E-14
Post	Lipid Metabolism	K01201	isotig10384	-1.73	9.76E-04
Post	Lipid Metabolism	K01634	isotig07786	2.68	7.58E-10
Post	Lipid Metabolism	K01641	isotig14068	-1.88	1.35E-07
Post	Lipid Metabolism	K01897	isotig13742	-2.21	3.97E-14
Post	Lipid Metabolism	K01897	isotig06457	1.03	2.48E-04
Post	Lipid Metabolism	K01897	isotig13685	-1.08	1.54E-11

Post	Lipid Metabolism	K04710	isotig11718	-2.14	4.41E-13
Post	Lipid Metabolism	K04712	isotig11364	1.12	9.95E-04
Post	Lipid Metabolism	K05309	isotig10936	-1.35	6.97E-06
Post	Lipid Metabolism	K07413	isotig15601	-4.12	4.65E-05
Post	Lipid Metabolism	K07424	isotig10014	1.73	4.11E-08
Post	Lipid Metabolism	K07508	isotig11546	1.16	1.71E-09
Post	Lipid Metabolism	K08743	isotig15068	4.78	1.36E-04
Post	Lipid Metabolism	K08764	isotig08223	-1.47	5.57E-10
Post	Lipid Metabolism	K10251	isotig11618	1.03	4.20E-05
Post	Lipid Metabolism	K10251	isotig15865	-3.14	2.80E-46
Post	Lipid Metabolism	K10257	isotig15015	-1.36	4.21E-06
Post	Lipid Metabolism	K10258	isotig15329	-2.26	1.88E-24
Post	Lipid Metabolism	K11155	isotig13916	-1.51	3.20E-09
Post	Lipid Metabolism	K11262	isotig03641	-2.82	1.28E-33
Post	Lipid Metabolism	K12309	isotig10126	-4.42	4.94E-06
Post	Lipid Metabolism	K12351	isotig15242	-1.27	6.13E-05
Post	Lipid Metabolism	K13356	isotig14072	-3.31	2.12E-08
Post	Lipid Metabolism	K13513	isotig15131	-1.66	3.14E-04
Post	Lipid Metabolism	K13516	isotig16734	-6.28	3.89E-10
Post	Lipid Metabolism	K13953	isotig17766	5.78	8.58E-08
Post	Other glycan degradation	K01201	isotig10400	-2.41	3.27E-14
Post	Other glycan degradation	K01201	isotig14368	-3.42	2.36E-05
Post	Other glycan degradation	K01201	isotig10384	-1.73	9.76E-04
Post	Other glycan degradation	K01206	isotig06593	-3.33	8.54E-14
Post	Other glycan degradation	K12309	isotig10126	-4.42	4.94E-06
Post	Other glycan degradation	K12311	isotig13439	-6.52	1.19E-11
Post	Other glycan degradation	K12373	isotig14029	-2.98	3.35E-15
Post	Pyruvate metabolism	K00026	isotig15407	-1.49	1.64E-04
Post	Pyruvate metabolism	K00128	isotig09962	-2.22	9.34E-15
Post	Pyruvate metabolism	K00162	isotig15346	-2.46	4.42E-05
Post	Pyruvate metabolism	K00626	isotig14729	-2.19	1.20E-20
Post	Pyruvate metabolism	K00627	isotig13822	-1.15	8.08E-05

Post	Pyruvate metabolism	K00873	isotig14289	-2.48	1.99E-20
Post	Pyruvate metabolism	K01610	isotig16257	4.56	4.01E-04
Post	Pyruvate metabolism	K01638	isotig10314	6.53	6.79E-12
Post	Pyruvate metabolism	K01895	isotig09700	6.1	2.49E-09
Post	Pyruvate metabolism	K11262	isotig03641	-2.82	1.28E-33
Post	Tyrosine metabolism	K00129	isotig14559	-4.27	2.26E-58
Post	Tyrosine metabolism	K00457	isotig09530	-3.84	4.68E-21
Post	Tyrosine metabolism	K00505	isotig13471	-2.12	1.62E-08
Post	Tyrosine metabolism	K00599	isotig16397	-2.41	7.18E-05
Post	Tyrosine metabolism	K00599	isotig11948	-1.99	4.55E-05
Post	Tyrosine metabolism	K00815	isotig13862	-3.87	1.23E-24
Post	Tyrosine metabolism	K01555	isotig03152	-1.22	4.54E-07
Post	Tyrosine metabolism	K13953	isotig17766	5.78	8.58E-08
Post	Tyrosine metabolism	K14455	isotig14718	-1.24	7.24E-05
Post	Valine, leucine and isoleucine degradation	K00022	isotig15495	-4.06	1.94E-70
Post	Valine, leucine and isoleucine degradation	K00128	isotig09962	-2.22	9.34E-15
Post	Valine, leucine and isoleucine degradation	K00253	isotig10260	-1.85	4.00E-05
Post	Valine, leucine and isoleucine degradation	K00626	isotig14729	-2.19	1.20E-20
Post	Valine, leucine and isoleucine degradation	K01641	isotig14068	-1.88	1.35E-07
Post	Valine, leucine and isoleucine degradation	K07508	isotig11546	1.16	1.71E-09
Post	Valine, leucine and isoleucine degradation	K09699	isotig11326	-2.39	4.31E-06
Post	Valine, leucine and isoleucine degradation	K11538	isotig15134	-3	8.97E-17
Post	Xenobiotics Biodegradation and Metabolism	K00022	isotig15495	-4.06	1.94E-70

Post	Xenobiotics Biodegradation and Metabolism	K00088	isotig06473	-1.24	7.37E-08
Post	Xenobiotics Biodegradation and Metabolism	K00128	isotig09962	-2.22	9.34E-15
Post	Xenobiotics Biodegradation and Metabolism	K00129	isotig14559	-4.27	2.26E-58
Post	Xenobiotics Biodegradation and Metabolism	K00485	isotig09806	-6.66	6.62E-24
Post	Xenobiotics Biodegradation and Metabolism	K00493	isotig14601	-4.94	6.67E-05
Post	Xenobiotics Biodegradation and Metabolism	K00599	isotig16397	-2.41	7.18E-05
Post	Xenobiotics Biodegradation and Metabolism	K00599	isotig11948	-1.99	4.55E-05
Post	Xenobiotics Biodegradation and Metabolism	K00626	isotig14729	-2.19	1.20E-20
Post	Xenobiotics Biodegradation and Metabolism	K00699	isotig05471	2.23	9.48E-10
Post	Xenobiotics Biodegradation and Metabolism	K01044	isotig08022	-2.89	4.84E-06
Post	Xenobiotics Biodegradation and Metabolism	K01101	isotig15153	-2.89	4.84E-06
Post	Xenobiotics Biodegradation and Metabolism	K01253	isotig03597	1.47	6.10E-06
Post	Xenobiotics Biodegradation and Metabolism	K01464	isotig14586	-1.54	2.15E-04
Post	Xenobiotics Biodegradation and Metabolism	K01555	isotig03152	-1.22	4.54E-07
Post	Xenobiotics Biodegradation and Metabolism	K01951	isotig13813	-1.9	2.05E-08
Post	Xenobiotics Biodegradation and Metabolism	K07413	isotig15601	-4.12	4.65E-05

Post	Xenobiotics Biodegradation and Metabolism	K07424	isotig10014	1.73	4.11E-08
Post	Xenobiotics Biodegradation and Metabolism	K07508	isotig11546	1.16	1.71E-09
Post	Xenobiotics Biodegradation and Metabolism	K13421	isotig14984	-1.26	5.12E-04
Post	Xenobiotics Biodegradation and Metabolism	K13953	isotig17766	5.78	8.58E-08
