# Supplementary material for: Comparative analysis of pre- and post-parasitic transcriptomes and mining pioneer effectors of Heterodera avenae
Source: Cell Biosci. 2017 Feb 14;7:11. doi: 10.1186/s13578-017-0138-6 (PMC5309974; doi:10.1186/s13578-017-0138-6)
Supplement: Supplementary file 8 — Additional file 8: Table S4. Putative phytonematode effectors identified in H. avenae. a Putative key genes based on blastx searching against other phytonematode effectors. b Carbohydrate-active enzymes with homology to those of other phytonematodes. * # CCN ESTs, number of ESTs in Cereal cyst nematode H. avenae. ‘Pre’ means significantly high expression in pre-parasitic stage; ‘Pre*’ means coding by singleton in pre-parasitic library. The singleton might be the gene with low expression and was sequenced only once. CBM, carbohydrate binding module; GH, glycoside hydrolase; GT, glycosyl transferase; PL, polysaccharide lysate. NR means non-redundant GenBank proteins database. [file 13578_2017_138_MOESM8_ESM.pdf]

Additional file 8: Table S4. Putative phytonematode effectors identified in *H. avenae.* (A) Putative key genes based on blastx searching against other phytonematode effectors. (B) Carbohydrate-active enzymes with homology to those of other phytonematodes. * # CCN ESTs, number of ESTs in Cereal cyst nematode *H. avenae*. 'Pre' means significantly high expression in pre-parasitic stage; 'Pre*' means coding by singleton in pre-parasitic library. The singleton might be the gene with low expression and was sequenced only once. CBM, carbohydrate binding module; GH, glycoside hydrolase; GT, glycosyl transferase; PL, polysaccharide lysate. NR means non-redundant GenBank proteins database.

Table S4A. Key genes as putative phytonematode effectors identified in *H. avenae* based on best blastx hits. Carbohydrate-active enzymes were not included in this list.

Accession	Description	Species	E value	Max identity	# CCN ESTs*
AEG64708	14-3-3 protein	*Heterodera glycines*	8.07E-168	99%	2
AIA08665	Annexin	*Heterodera avenae*	8.83E-152	67%	1
AAN32888	Annexin 4C10	*Heterodera glycines*	6.60E-14	56%	1
AAC47533	Calponin homolog MjCAP-1	*Meloidogyne javanica*	3.43E-43	66%	1
AFK76483	Calreticulin	*Radopholus similis*	1.00E-157	87%	1
ABC88426	Cathepsin D-like aspartic proteinase preproprotein	*Meloidogyne incognita*	1.78E-37	82%	4
ACJ13100	Cathepsin L	*Heterodera avenae*	0	91%	1
AAS84611	Cathepsin L-like cysteine proteinase I variant form precursor	*Heterodera glycines*	1.46E-90	80%	1
AGL80530	Cathepsin-S	*Heterodera avenae*	7.58E-128	96%	1
D1FNJ9	CLAVATA3/ESR (CLE)-related protein 4A-1 precursor	*Globodera rostochiensis*	7.00E-04	37%	1
D1FNK3	CLAVATA3/ESR (CLE)-related protein 4B-2 precursor	*Globodera rostochiensis*	0.006	42%	1
ADD62691	Cellulose binding protein	*Heterodera avenae*	1.33E-13	33%	1
AAN32887	Cellulose binding protein	*Heterodera glycines*	9.40E-18	32%	1
AAK83075	Collagen	*Meloidogyne javanica*	5.36E-13	79%	1
AAB61596	Galectin	*Globodera rostochiensis*	1.20E-92	83%	1
AMQ99047	Fatty acid and retinol binding protein 1	*Heterodera avenae*	1.21E-125	100%	1
AMQ99048	Fatty acid and retinol binding protein 2	*Heterodera avenae*	2.53E-147	100%	1
AAC46496	SEC-1	*Meloidogyne incognita*	1.68E-42	32%	1
CAA70477	SEC-2 proteins	*Globodera pallida*	5.65E-49	53%	1
ABN64198	Glutathione S-transferase-1	*Meloidogyne incognita*	1.53E-81	56%	5
CAB48391	Peroxiredoxin	*Globodera rostochiensis*	1.16E-79	86%	3
ACZ67203	Peroxiredoxin	*Meloidogyne incognita*	2.80E-31	47%	1
CAD38524	Putative glutathione peroxidase	*Globodera rostochiensis*	1.50E-99	85%	7
CAD38523	Secreted glutathione peroxidase	*Globodera rostochiensis*	7.55E-165	88%	5
AAR35032	SXP/RAL-2 protein	*Meloidogyne incognita*	5.05E-30	47%	1
CAM84511	Transthyretin-like protein 2 precursor	*Radopholus similis*	1.99E-33	84%	2
CAM84512	Transthyretin-like protein 3 precursor	*Radopholus similis*	1.01E-24	81%	1
**CAM84513**	Transthyretin-like protein 4 precursor	*Radopholus similis*	1.99E-76	73%	1
**AAW56830**	Troponin C-like protein	*Meloidogyne incognita*	2.32E-112	97%	2
**AAN32889**	Ubiquitin extension protein	*Heterodera glycines*	7.35E-57	85%	2
**AAP30081**	Ubiquitin extension protein	*Heterodera schachtii*	5.36E-30	55%	2
**AAK60209**	Venom allergen-like protein 1	*Heterodera glycines*	3.98E-87	75%	2
**AAK55116**	Venom allergen-like protein 2	*Heterodera glycines*	1.87E-105	66%	1
**AAL40718**	Myosin regulatory light chain	*Meloidogyne incognita*	1.35E-20	80%	2
**CAB75701**	Putative hypodermis secreted protein	*Globodera rostochiensis*	1.80E-97	66%	1
**ADI82806**	Dorsal esophageal gland-specific protein	*Heterodera avenae*	0	100%	1
**AAN08587**	Putative esophageal gland cell secretory protein 21	*Meloidogyne incognita*	3.49E-117	48%	2
**AAN15808**	Putative esophageal gland cell secretory protein 28	*Meloidogyne incognita*	3.62E-60	61%	3
**AAN15809**	Putative esophageal gland cell secretory protein 29	*Meloidogyne incognita*	1.29E-24	46%	1
**AAQ10017**	Putative esophageal gland cell secretory protein 3	*Meloidogyne incognita*	5.52E-32	49%	2

**AAM95699**	Putative esophageal gland cell secretory protein 33A09	*Heterodera glycines*	9.76E-24	35%	4
**AAP30755**	Putative esophageal gland cell secretory protein 29D09	*Heterodera glycines*	2.88E-29	37%	1
**AAP30760**	Putative esophageal gland cell secretory protein G10A07	*Heterodera glycines*	1.78E-08	74%	1
**AAP30754**	Putative esophageal gland cell secretory protein G11A06	*Heterodera glycines*	2.06E-42	61%	1
**AAO85452**	Putative esophageal gland cell secretory protein G12H04	*Heterodera glycines*	4.37E-67	67%	3
**AAO85455**	Putative esophageal gland cell secretory protein G17G06	*Heterodera glycines*	6.19E-15	53%	1
**AAO85457**	Putative esophageal gland cell secretory protein G19B10	*Heterodera glycines*	1.85E-24	33%	3
**AAO85459**	Putative esophageal gland cell secretory protein G20E03	*Heterodera glycines*	1.16E-93	73%	5
**AAM50038**	Putative esophageal gland cell secretory protein G27D09	*Heterodera glycines*	6.83E-11	43%	1
**AAP30774**	Putative esophageal gland cell secretory protein G30E03	*Heterodera glycines*	8.01E-74	64%	10
**AAP30775**	Putative esophageal gland cell secretory protein G32E03	*Heterodera glycines*	8.04E-13	90%	7
**AAP30762**	Putative esophageal gland cell secretory protein G7E05	*Heterodera glycines*	8.07E-65	84%	3
**AAN32892**	Putative esophageal gland cell secretory protein 4D06	*Heterodera glycines*	3.38E-61	60%	20
**AAN32891**	Putative esophageal gland cell secretory protein 5D06	*Heterodera glycines*	2.96E-20	45%	1
**AAN32886**	Putative esophageal gland cell secretory protein G2D01	*Heterodera glycines*	3.21E-49	67%	1
**AAO33473**	Putative esophageal gland cell secretory protein 4E02	*Heterodera glycines*	2.05E-31	79%	3
**AAO33477**	Putative esophageal gland cell secretory protein 4G05	*Heterodera glycines*	4.56E-19	58%	2
**AAO33475**	Putative esophageal gland cell secretory protein 5D08	*Heterodera glycines*	5.79E-37	60%	1
**AAF76926**	Putative esophageal gland cell secretory protein SYV55	*Heterodera glycines*	6.34E-102	92%	2

* # CCN ESTs, number of ESTs in Cereal cyst nematode *H. avenae*.

Table S4B. The CAZy enzymes identified in the transcriptome of *H. avenae* with homology to that of other phytonematodes.

CCN enzyme	CAZy family	Best identity descriptor in NR	Accession	E value	Expression bias
ISOTIG18265	CBM2	Expansin [*Heterodera glycines*]	ADL29728	1.37E-96	-
ISOTIG19070	CBM2	Expansin [*Heterodera glycines*]	ADL29728	3.53E-18	Pre
ISOTIG16975	CBM2	Expansin B1 [*Heterodera avenae*]	AEP19215	0	Pre
ISOTIG18443	CBM2	Expansin B3, partial [*Globodera pallida*]	AEU04794	5.25E-88	-
ISOTIG16488	GH5	Beta-1,4-endoglucanase-2 precursor [*Heterodera glycines*]	AAC48326	3.91E-93	Pre
HO9SJLY04JO3RG_3	GH5	Cellulase [*Heterodera glycines*]	AAM50039	1.24E-63	Pre*
ISOTIG15773	GH5	Cellulase ENG-5 [*Heterodera glycines*]	AAN32884	1.21E-164	Pre
ISOTIG15246	GH5	Cellulase, partial [*Aphelenchoides fragariae*]	AFI63769	5.12E-57	-
ISOTIG17481	GH5	GHF5 endo-1,4-beta-glucanase precursor [*Radopholus similis*]	ABV54446	1.52E-80	Pre
ISOTIG17176	GH5	Beta-1,4-endoglucanase 3 [*Heterodera avenae*]	AFQ55682	0	Pre
ISOTIG16403	GH5	Beta-1,4-endoglucanase 2 [*Heterodera avenae*]	AFQ55680	0	Pre
ISOTIG14720	GH5/ CBM2	Beta-1,4-endoglucanase 1 [*Heterodera avenae*]	ACO55952	0	Pre
ISOTIG15098	GH5/ CBM2	Beta-1,4-endoglucanase precursor [*Globodera rostochiensis*]	AAC63988	1.07E-166	Pre
ISOTIG08169	GT20	Putative trehalose 6-phosphate synthase [*Aphelenchus avenae*]	CAH18869	0	-
ISOTIG18756	PL3	Pectate lyase 1 [*Heterodera avenae*]	ADD82848	3.68E-33	Pre
HOWVQCB02G9WZE_10	PL3	Pectate lyase 2, partial [*Heterodera avenae*]	ADD73447	1.31E-49	Pre*
HOWVQCB02JPONP_5	PL3	Pectate lyase [*Heterodera glycines*]	ADW77533	2.20E-72	Pre*
HOWVQCB02GVIDZ_7	PL3	pectate lyase [*Heterodera glycines*]	ADW77534	9.46E-115	Pre*

'Pre' means significantly high expression in pre-parasitic stage; 'Pre*' means coding by singleton in pre-parasitic library. The singleton might be the gene with low expression and was sequenced only once. CBM, carbohydrate binding module; GH, glycoside hydrolase; GT, glycosyl transferase; PL, polysaccharide lysate. NR means non-redundant GenBank proteins database.
